# Fibrosis Development Linked to Alterations in Glucose and Energy Metabolism and Prooxidant–Antioxidant Balance in Experimental Models of Liver Injury

**DOI:** 10.3390/antiox12081604

**Published:** 2023-08-12

**Authors:** Dmitry S. Semenovich, Nadezda V. Andrianova, Ljubava D. Zorova, Irina B. Pevzner, Polina A. Abramicheva, Andrey V. Elchaninov, Olga V. Markova, Aleksandra S. Petrukhina, Dmitry B. Zorov, Egor Y. Plotnikov

**Affiliations:** 1 A.N. Belozersky Institute of Physico-Chemical Biology, Moscow State University, 119992 Moscow, Russia; 2 V.I. Kulakov National Medical Research Center for Obstetrics, Gynecology and Perinatology, Ministry of Healthcare of Russian Federation, 117198 Moscow, Russia; 3 Avtsyn Research Institute of Human Morphology of Federal State Budgetary Scientific Institution “Petrovsky National Research Centre of Surgery”, 117418 Moscow, Russia; 4 K.I. Skryabin Moscow State Academy of Veterinary Medicine and Biotechnology, 109472 Moscow, Russia

**Keywords:** liver fibrosis, bile duct ligation, hepatic ischemia, glycolysis, Krebs cycle, mitochondrial respiration, oxidative stress, glutathione, metabolism

## Abstract

The development of liver fibrosis is one of the most severe and life-threatening outcomes of chronic liver disease (CLD). For targeted therapy of CLD, it is highly needed to reveal molecular targets for normalizing metabolic processes impaired in damaged liver and associated with fibrosis. In this study, we investigated the morphological and biochemical changes in rat liver models of fibrosis induced by chronic administration of thioacetamide, carbon tetrachloride, bile duct ligation (BDL), and ischemia/reperfusion (I/R), with a specific focus on carbohydrate and energy metabolism. Changes in the levels of substrates and products, as well as enzyme activities of the major glucose metabolic pathways (glycolysis, glucuronidation, and pentose phosphate pathway) were examined in rat liver tissue after injury. We examined key markers of oxidative energy metabolism, such as the activity of the Krebs cycle enzymes, and assessed mitochondrial respiratory activity. In addition, pro- and anti-oxidative status was assessed in fibrotic liver tissue. We found that 6 weeks of exposure to thioacetamide, carbon tetrachloride, BDL or I/R resulted in a decrease in the activity of glycolytic enzymes, retardation of mitochondrial respiration, elevation of glucuronidation, and activation of pentose phosphate pathways, accompanied by a decrease in antioxidant activity and the onset of oxidative stress in rat liver. Resemblance and differences in the changes in the fibrosis models used are described, including energy metabolism alterations and antioxidant status in the used fibrosis models. The least pronounced changes in glucose metabolism and mitochondrial functions in the I/R and thioacetamide models were associated with the least advanced fibrosis. Ultimately, liver fibrosis significantly altered the metabolic profile in liver tissue and the flux of glucose metabolic pathways, which could be the basis for targeted therapy of liver fibrosis in CLD caused by toxic, cholestatic, or I/R liver injury.

## 1. Introduction

Liver diseases belong to a global health problem, resulting in approximately 2 million deaths worldwide annually [1]. There are numerous causes eventually resulting in chronic liver disease (CLD), including viral attacks, alcoholic addiction, autoimmune and genetic diseases [2]. The high mortality in these pathological conditions is due to complications, such as chronic portal hypertension, ascites, hemorrhage, hepatic encephalopathy, and hepatorenal syndrome [3]. 

The main pathogenetic mechanism of CLD, leading to a variety of complications and complicates therapy, involves the onset of fibrosis [4]. Being a result of pathogenic intervention, hepatocyte death occurs, in some cases accompanied by the infiltration of immune cells and the development of inflammation. These processes activate Kupffer cells and the transdifferentiation of hepatic stellate cells (HSCs) into liver myofibroblasts, which are responsible for the progressive formation of extracellular matrix (ECM) in the liver, forming scar tissue that can neither function nor repair itself. 

Liver fibrogenesis is a highly dynamic process that opens up possibilities for therapy of this pathological condition [5]. Recently, energy metabolism—particularly glycolysis—has been frequently proposed as a target for the treatment of fibrosis, since it has been known that fibroblasts involved in the formation of fibrotic scar tissue predominantly use glycolysis as an energy source and are characterized by enhanced glycolytic pathway [6,7]. However, the role of glycolysis in the development of liver fibrosis is poorly described, and the differences in the pathogenesis of the various liver diseases raise the question: how common are changes in energy metabolism associated with fibrosis? 

The aim of this work was to describe the changes in glucose metabolism and energy flux observed in the development of liver fibrosis of different etiologies. We compared the changes observed in liver fibrosis induced by thioacetamide (TAA) and carbon tetrachloride (CCl_4_) as well as after bile duct ligation (BDL) and ischemia/reperfusion (I/R). In these models, liver tissue was damaged in different ways, which may lead to various effects on liver cell metabolism during fibrosis development. 

## 2. Materials and Methods

### 2.1. Chemicals

Reagents were obtained as follows: Tris base, 3-(N-Morpholino)propanesulfonic acid (MOPS), bovine serum albumin (BSA) fatty acids free, cytochrome c from equine heart, chloramine-T trihydrate, p-dimethylaminobenzaldehyde (Ehrlich’s reagent), D-erythrulose, 4-hydroxyproline (Hyp), coenzyme A (CoA) lithium salt, acetyl coenzyme A lithium salt, carbonyl cyanide 3-chlorophenylhydrazone (CCCP), 70% perchloric acid, phenazine methosulfate (PMS), rotenone; sucrose, β-nicotinamide adenine dinucleotide oxidized (NAD) and reduced (NADH), glycogen from bovine liver, glutathione reductase and glucose-6-phosphate dehydrogenase from baker’s yeast, sodium α-ketoglutarate, sodium succinate, and sodium azide from Sigma-Aldrich (Merck, Darmstadt, Germany); ethylene glycol bis(2-aminoethylether)-N,N,N’,N’-tetra acetic acid (EGTA), glycerol and N,N,N’,N’-tetramethyl-ethylenediamine (TEMED) from Serva (Heidelberg, Germany); 2-thiobarbituric acid (TBA), reduced glutathione (GSH), 5,5′-dithio-bis-(2-nitrobenzoic acid) (Ellman’s reagent), β-nicotinamide adenine dinucleotide phosphate oxidized (NADP), reduced (NADPH), trichloroacetic acid (TCA), and 2,4-dinitrophenylhydrazine from AppliChem GmbH (Darmstadt, Germany); D-ribose-5-phosphate barium salt, D-Fructose-1,6-bisphosphate calcium salt, lactate dehydrogenase (LDH) from pig muscle, iodonitrotetrazolium chloride (INT), pyruvate kinase from rabbit muscle, alcohol dehydrogenase from horse liver, glyceraldehyde-3-phosphate dehydrogenase (GAPDH), and aldolase from rabbit muscle from Reanal (Budapest, Hungary); oxaloacetic acid, thiamine pyrophosphate, adenosine 5′-diphosphate (ADP), adenosine 5′-triphosphate (ATP), D-glucose-6-phosphate sodium salt, D-Fructose-6-phosphate disodium salt, and uridine 5′-diphosphoglucuronic acid trisodium salt from Santa Cruz Biotechnology (Dallas, TX, USA); benzidine and oxalacetic acid from Chem-Impex International (Wood Dale, IL, USA); uridine 5′-diphosphoglucose from MT Bio-Tech Co. (Changsha, China). All reagents were of analytical grade, HPLC grade, or the best available pharmaceutical grade. All solutions were prepared using water purified by the Milli-Q system.

### 2.2. Animals

In experiments, 4–6-month-old, 280–320 g male outbred Wistar rats (n = 32) were used. The number of animals in each experimental group used for specific methods is presented in Supplementary Table S1. All procedures were performed in accordance with the *Animal Research: Reporting of In Vivo Experiments* (ARRIVE) guidelines. Rats were treated according to animal protocols evaluated and approved by the animal ethics committee of the A.N. Belozersky Institute of Physico-Chemical Biology Lomonosov Moscow State University: Protocol 115-15/09/2021 from 3 September 2021. Animals had unlimited access to food and water and were kept in cages in a temperature-controlled environment (20 ± 2 °C) under the 12/12 h light/dark regime.

### 2.3. Modeling of Liver Damage in Animals

**Figure 1:**
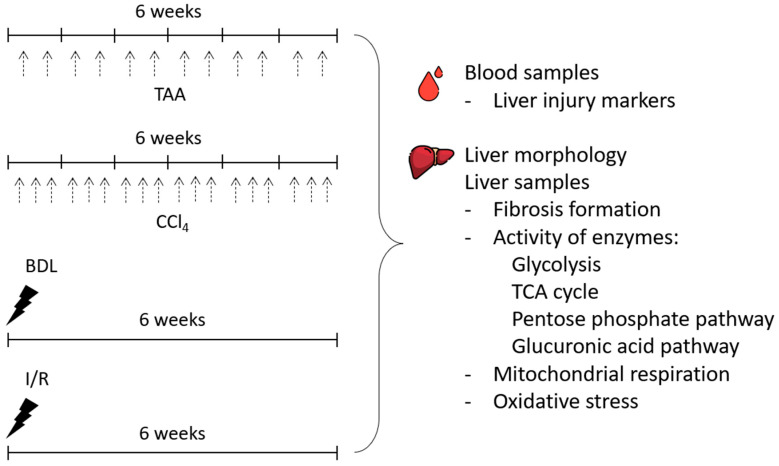
Experimental design. Experiments were performed on 4 different liver fibrosis models, after administration of TAA or CCl_4_ (each administration is indicated by black dashed arrows) and after BDL or I/R.

In the study, the severity of liver injury and morphologic changes after 6 weeks of modeling liver lesions of different origins (Figure 1) was explored. To simulate toxic liver injury and the development of liver fibrosis, rats were injected intraperitoneally with TAA (200 mg/kg) twice weekly for 6 weeks. Control animals were injected intraperitoneally with saline (5 mL/kg, 2 times a week for 6 weeks). In another group, CCl_4_, in the form of a 20% solution in petrolatum oil (1.0 mL/kg), was administered to rats intragastrically 3 times a week for 6 weeks. Control animals were administered with petrolatum oil intragastrically (1 mL/kg, 3 times a week for 6 weeks).

For modeling BDL, rats were intraperitoneally administrated with chloral hydrate (300 mg/kg), followed by a midline laparotomy and the muscle wall transection. Through the incision, the duodenal loop was brought to the surgical wound and the common bile duct was isolated. Then, a ligature was applied to the proximal and then to the distal part of the bile duct, and the duct was cut between the ligatures. The surgical wound was sutured with silk layer by layer.

For I/R modeling, the rats underwent midline laparotomy followed by abdominal wall transection, and the medial, left, and right lobes of the liver were brought to the surgical wound. Then, the hepatic portal triad, including the portal vein, hepatic artery and common bile duct, was isolated. A vascular clamp was applied to the hepatic triad for 45 min. The body temperature of the rats during ischemia was maintained at 37 °C. Then, the clamp was removed and the occurrence of reperfusion was monitored by the color changes of the liver lobes exposed to ischemia. At the end, the surgical wound was sutured with polyglycolide.

Intact rats were used as a control group because no differences were observed between intact rats and rats treated with saline or petrolatum oil and sham-operated animals (Supplementary Figure S1).

### 2.4. Sampling of Liver Tissue and Isolation of Subcellular Fractions

The animals were humanely euthanized according to accepted bioethical requirements. Liver tissue was collected, immersed in cold isolation medium (250 mM sucrose, 10 mM MOPS, 0.1% BSA, 1 mM EGTA, pH 7.2), and used to isolate mitochondrial, microsomal, and cytosolic fractions by differential centrifugation. Liver mitochondria were isolated according to the method of Johnson and Lardy in our modification [8]. Briefly, liver tissue was homogenized in an isolation medium at a ratio of 1:9 (*w*/*v*). The homogenate was centrifuged at 1000× *g* for 5 min and the supernatant was centrifuged at 12,500× *g* for 10 min at 4 °C. The resultant mitochondrial pellet was washed twice in cold isolation medium and resuspended in the isolation medium for final protein concentration of 40–60 mg/mL. The supernatant obtained after pelleting of mitochondria was centrifuged at 105,000× *g* for 60 min at 4 °C to obtain a cytosolic fraction and a microsome pellet. The microsome pellet was resuspended in a medium containing 20 mM MOPS pH 7.2, 1 mM EDTA, and 20% glycerol. The content of total protein in the subcellular fractions was determined by the BCA method using a commercial kit (Sigma, Burlington, MA, USA). Aliquots of the subcellular fractions were stored at −70 °C.

### 2.5. Biochemical Analysis of the Blood

Blood was placed in tubes containing a coagulation activator and a separating gel and allowed to stand at room temperature for 20 min. The serum was separated by centrifugation at 1500× *g* for 10 min and stored at −70 °C. The activity of liver marker enzymes in serum (AST, ALT, ALP, and GGT) and the concentration of total protein, albumin, and total bilirubin were determined spectrophotometrically using commercial reagent kits according to the manufacturer’s instructions (Olvex Diagnosticum, Saint Petersburg, Russia).

### 2.6. Morphological Studies and Hydroxyproline Measurement

The extracted liver was weighed and photographed. The relative weight was calculated as the ratio of the liver weight to the body weight of the rat (expressed as %).

Liver samples were fixed in 10% buffered formalin and embedded in paraffin according to the standard protocol. Paraffin sections of 5 µm were prepared and stained using a commercial kit (Biovitrum, Saint Petersburg, Russia) according to the Mallory method. Stained liver sections were examined under an Axio Scope A1 microscope (Carl Zeiss, Oberkochen, Germany) with an MRc.5 camera (Carl Zeiss, Oberkochen, Germany). Sections were examined in a blind fashion for the presence of fibrosis.

To assess the development of liver fibrosis, the measurement of the hydroxyproline content in the tissue was also performed. The method is based on the alkaline hydrolysis of tissue and subsequent quantification of free hydroxyproline (Hyp) in the hydrolysates [8]. Liver tissue (20 mg) was added to 0.5 mL of 7 M KOH and hydrolyzed at 120 °C for 40 min. The hydrolysate was neutralized with 3.5 M of H_2_SO_4_ and mixed with a buffered chloramine T reagent. Then the Ehrlich reaction was performed according to the standard protocol and the absorption of the solution at 550 nm was determined using a PE-5400UV spectrophotometer (“Ekrokhim” LLC, Saint Petersburg, Russia).

### 2.7. Determination of Substrates and Products of Glucose Metabolism

To determine the substrates and products of glucose metabolism, liver homogenates were prepared at 1:4 (*w*/*v*) in 6% HClO_4_ and centrifuged at 12,000× *g* for 15 min at 4 °C. The supernatant was neutralized with 5 M of K_2_CO_3_ to reach pH 7, stored in an ice bath for 15 min, and then centrifuged. In addition, studies were performed with whole blood. For this, heparinized blood was mixed with 6% HClO_4_ at a ratio of 1:1 (*v*/*v*) and treated in the same manner as described above. The supernatants were used for further analyzes.

All analyses were performed by spectrophotometric methods. Glucose and lactate concentrations were determined by the glucose oxidase method based on the Trinder reaction using commercial kits (Olvex Diagnosticum, Saint Petersburg, Russia). Pyruvate concentration was determined by the enzymatic method using lactate dehydrogenase [9]. The content of glucose-6-phosphate was determined by enzymatic protocol with glucose-6-phosphate dehydrogenase from baker’s yeast [10]. Absorption measurements were carried out on a PE-5400 UV (Ekrokhim) spectrophotometer.

The total level of pentoses was determined by the reaction with orcinol [11] with modifications. Perchlorate liver extract (1:4, *w*/*v*) was mixed 1:1 (*v*/*v*) with orcinol reagent containing 1% orcinol, 0.1% FeCl_3_-6H_2_O, and 35% HCl and boiled for 40 min. The absorbance was determined at 620 nm on an INNO plate spectrophotometer (LTEK Co., Ltd., Instruments, Seongnam-si, Republic of Korea). Calibration was performed with D-ribose-5-phosphate in the concentration range of 0.05–0.5 mM.

The total level of glucuronic acid in neutralized perchlorate liver extract was determined by the spectrophotometric method in our modification [12]. A sample was mixed with 0.1 M of a sodium acetate buffer, pH 5.0, and 0.6% benzidine (prepared with glacial acetic acid) at a ratio of 3:1:3 (*v*/*v*). Samples were boiled for 15 min, cooled in an ice bath, and absorbance was determined at 410 nm on an INNO plate spectrophotometer.

The determination of glycogen content in the liver was performed by the colorimetric phenol-sulfuric acid method [13]. 

### 2.8. Determination of the Activity of Glucose Metabolism Enzymes

The activity of the enzymes of glycolysis and the pentose phosphate pathway (PPP) was determined in the cytosolic fraction, and the activity of the enzymes of the glucuronic pathway was determined in the microsomal fraction of the liver. The activities of hexokinase (HK, EC 2.7.1.1) and glucokinase (GK, EC 2.7.1.2) [14], glucose-6-phosphate isomerase (GPI, EC 5.3.1.9) [15], glyceraldehyde-3-phosphate dehydrogenase (GAPDH, EC 1.2.1.12) [16], phosphofructokinase (PFK, EC 2.7.1.11) [17], 3-phosphoglycerate kinase (PGK, EC 2.7.2.3) [18], pyruvate kinase (PK, EC 2.7.1.40) [19], lactate dehydrogenase (LDH, EC 1.1.1.27) [20], glucose-6-phosphate dehydrogenase (G6PDH, EC 1.1.1.49) and 6-phosphogluconate dehydrogenase (6PGDH, EC 1.1.1.44) [21], transketolase (TK, EC 2.2.1.1) [22], UDP-glucose dehydrogenase (UGDH, EC 1.1.1.22) [23] were determined by kinetic methods in coupled enzymatic reactions by absorbance of the formed NAD(P)H at 340 nm. Absorbance was measured in the “kinetics” mode at a temperature of 37 °C in a 96-well plate on a Zenit 3100 plate reader. As 1 unit (U) of enzyme activity, 1 nmol of NAD(P)H formed (or consumed) in 1 min at 37 °C was taken. The activity of UDP-glucuronosyltransferase (UGT, EC 2.4.1.17) was determined spectrophotometrically using 4-nitrophenol as an acceptor [24]. Aldolase activity was determined by a colorimetric method based on the reaction of 2,4-dinitrophenylhydrazine with phosphotrioses in an alkaline medium [25]. As 1 unit of aldolase activity (U), 1 nmol of phosphotriose, formed from fructose-1,6-bisphosphate in 1 min at 37 °C, was taken.

### 2.9. Determination of PDHC and Krebs Cycle Enzymes Activity

To determine the activity of the PDHC and Krebs cycle enzymes, mitochondria were sonicated in an ice bath (6 cycles, each by 10 s). The activity of PDHC (EC 1.2.4.1) was determined by the reduction of NAD recorded at 340 nm [26]. The activity of citrate synthase (CS, EC 2.3.3.16) was determined by a kinetic method based on the nonenzymatic reaction of coenzyme A (CoA-SH) with Ellman’s reagent [27]. The activity of NADP-dependent isocitrate dehydrogenase (ICDH, EC 1.1.1.42) was determined by a kinetic method based on the absorption of NADPH [28]. The activity of α-ketoglutarate dehydrogenase (α-KGDH, EC 1.2.4.2) and succinate dehydrogenase (SDH, EC 1.3.5.1) was assessed using potassium ferricyanide and iodonitrotetrazolium chloride as electron acceptors, respectively [29,30]. The activity of malate dehydrogenase (MDH, EC 1.1.1.37) was determined by the kinetic method through measuring formed NADH [31]. Absorbance was measured using the Zenith 3100 plate reader in the “kinetics” mode at 37 °C in a 96-well plate. As 1 unit (U) of activity of ICDH and MDH, 1 nmol of NAD (P)H formed (or consumed) in 1 min at a temperature of 37 °C was taken. As 1 unit (U) of α-KGDH activity, 1 nmol of reduced potassium ferricyanide was taken in 1 min at a temperature of 37 °C. As 1 unit (U) of SDH activity, 1 nmol of INT-formazan, formed in 1 min at a temperature of 37 °C, was taken.

### 2.10. Assessment of Respiration of Mitochondria and Cytochrome C Oxidase Activity

Mitochondrial respiration was measured by a polarographic method using the Strathkelvin SI782 Precision Dissolved Oxygen Respirometer (Cole-Parmer, Vernon Hills, IL, USA). The incubation medium contained 250 mM of sucrose, 10 mM of MOPS, 2.5 mM of KH_2_PO_4_, 2.5 mM of MgCl_2_, 1.0 mM of EGTA, and a pH 7.2. The experiment was performed at 25 °C using 5 mM sodium succinate as a substrate in the presence of 2 μM rotenone. The concentration of mitochondrial protein in the sample was 1.0 mg/mL. State 2 corresponds to respiration in the presence of a substrate (succinate) (V_2_). The mitochondrial respiration rate in state 3 (V_3_) was recorded after the addition of 100 μM of ADP. The uncoupled respiration rate (V_CCCP_) was recorded in the presence of 0.1 μM of CCCP. Mitochondrial coupling (respiratory control) was determined as the ratio of V_3_/V_4_. Oxygen consumption curves were obtained and processed using the “Strathkelvin Oxygen System. Data Analysis Module” program version 3.0.1.16 (Strathkelvin Instruments, North Lanarkshire, Scotland).

Cytochrome *c* oxidase (COX) activity in mitochondria was determined by the oxidation rate of reduced cytochrome by absorbance at 550 nm [28]. Mitochondria were preliminarily sonicated in an ice bath (6 cycles of 10 s) and COX activity was estimated using the coefficient of extinction for reduced cytochrome *c* 18.5 mM^−1^ cm^−1^. Absorption measurements were carried out on an INNO plate spectrophotometer (Cheongju-si, Republic of Korea).

### 2.11. Determination of Oxidative Stress in Liver Tissue

The content of 2-thiobarbituric acid-reactive substances (TBARS) in liver homogenate was determined after incubation with 50 μM of FeSO_4_ and 0.5 mM of ascorbate at 37 °C for 1 h. Samples were boiled with TBA reagent, cooled, and centrifuged at 3000× *g* for 10 min. The absorbance at 532 nm was determined in the supernatant. The TBARS content was estimated from an extinction coefficient of 156 mM^−1^ cm^−1^ [32]. 

The level of reduced glutathione (GSH) in liver tissue was determined spectrophotometrically using Ellman reagent at 412 nm. Glutathione content was estimated using an extinction coefficient of 14.150 mM^−1^ cm^−1^ [33]. 

The activity of glutathione peroxidase (GPx, EC 1.11.1.9) was determined by the spectrophotometric kinetic method using hydrogen peroxide as substrate. Measurements were performed at 340 nm on a Zenith 3100 plate reader at a temperature of 37 °C [34].

### 2.12. Real-Time PCR

The mRNA expression of the enzymes of the glucuronic acid pathway (UGDG and UGT) was determined by RT-PCR. Liver tissue samples were snap frozen in liquid nitrogen and stored at −80 °C. The RNA Solo Kit (Evrogen, Moscow, Russia) was used for nucleic acid isolation and treatment with duplex-specific nuclease. After RNA extraction, the concentration was determined using Biophotometer (Eppendorf, Framingham, MA, USA). Reverse transcription was performed using the MMLV RT kit (Evrogen, Moscow, Russia). The RT-PCR was performed with the Bio-Rad Real-Time PCR System (Bio-Rad, California, Hercules, CA, USA) using SYBR Green as a double-stranded DNA-specific binding dye. Amplification was performed using 5× qPCRmix-HS mastermix (Evrogen, Moscow, Russia). Primers (Table 1) were selected using the Beacon Designer 7 program (Premier Biosoft Int., San Francisco, CA, USA) and by using the NCBI and Ensemble search databases. The primers were synthesized by DNA-Synthesis (Moscow, Russia). Their efficiency was calculated by generating a standard curve for each target gene using a five-fold serial dilution of the cDNA pool and ranged from 1.8–2.0. mRNA expression levels were calculated as E^–Ct^, where E is primer efficiency and Ct is the cycle number at which product fluorescence increased above threshold.

### 2.13. Western Blot Analysis

Rat liver tissue samples were homogenized in cold 1× PBS, a pH 7.4 with 1 mM of phenylmethylsulfonyl fluoride at 4 °C. The homogenate was centrifuged at 3000× *g* for 3 min, and the supernatant was mixed with 4× sample buffer containing 10% 2-mercaptoethanol and boiled for 5 min. Protein concentration was measured using a bicinchoninic acid assay (Sigma, Burlington, MA, USA). Total protein load was also monitored by Stain-Free imaging according to the manufacturer’s instructions (Bio-Rad, Hercules, CA, USA). All Western blot bands were normalized to the intensity of the same samples in Stain-Free Imaging. Liver samples were loaded onto a 15% Tris-glycine polyacrylamide gel (10 µg total protein per lane). After electrophoresis, the gels were transferred onto PVDF membrane (Amersham Pharmacia Biotech, Buckinghamshire, United Kingdom). Membranes were blocked with 5% (*w*/*v*) nonfat milk (SERVA, Heidelberg, Germany) in PBS containing 0.05% Tween-20 (Panreac, Barcelona, Spain) and then incubated with the primary antibodies anti-GAPDH 1:1000 (MAA785Ra21, Cloud Clone Inc., Wuhan, Hubei, PRC). Membranes were then incubated with secondary anti-mouse IgG antibodies conjugated with horseradish peroxidase 1:7500 (Imtek, Moscow, Russia). Detection was performed using the WesternBright Enhanced Chemiluminescence Kit (Advansta, San Jose, CA, USA) with the ChemiDoc MP Imaging System (Bio-Rad, Hercules, CA, USA).

### 2.14. Bioinformatics Analysis

Principal component analysis was performed using MetaboAnalyst 5.0 software. Hierarchical clustering analysis was performed using Heatmapper (http://heatmapper.ca/, accessed on 13 June 2023). The Pearson distance algorithm for similarity measurement and the complete linkage clustering algorithm for clustering were selected. 

### 2.15. Statistical Analysis

Statistical analysis was performed with the GraphPad Prism 7 (GraphPad Software Inc., San Diego, CA, USA). The data was analyzed by parametric one-way ANOVA with Sidak’s multiple comparison test. The outliers were identified by Grubbs’ test and excluded. The results are presented as mean ± SEM with *p* < 0.05 considered as statistically significant, * *p* < 0.05, ** *p* < 0.01, *** *p* < 0.001.

## 3. Results

### 3.1. Morphological and Biochemical Markers of Liver Damage

**Figure 2:**
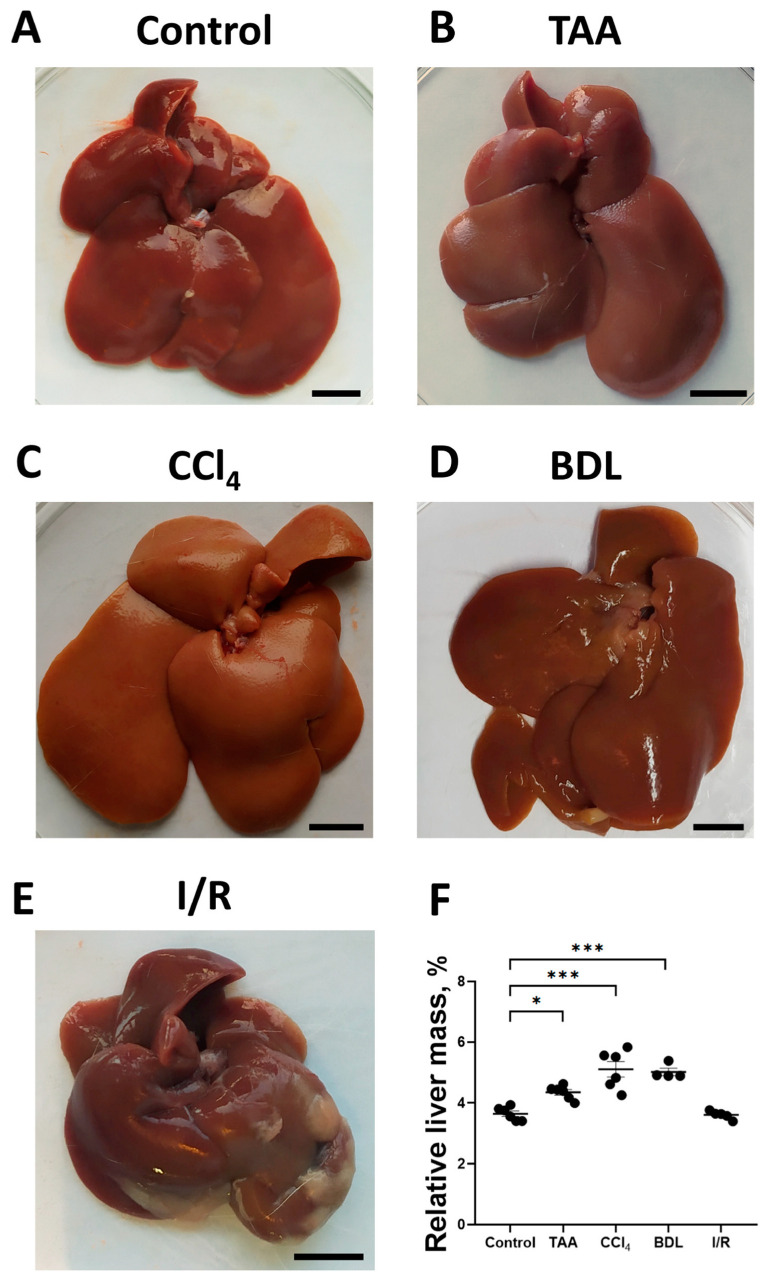
Morphological characteristics of the liver in experimental models of fibrosis. (**A**–**E**) Macroscopic images of the liver of control rats and of rats 6 weeks after administration of TAA, CCl_4_ or modeling BDL and I/R, respectively. (**F**) Relative liver weight 6 weeks after modeling injury of different origin. Bar—1 cm. The number of rats n ≥ 4 in all experimental groups. * *p* < 0.05, *** *p* < 0.001 (one-way ANOVA).

To assess the development of fibrosis in the liver, morphological examination was performed, and the relative liver mass was measured after euthanasia (Figure 2A–D). In the rats of the TAA group, an increase in organ size, tissue compaction, formation of a pronounced granularity on the liver surface with individual small micro-nodules and rounding of the margins were observed as compared with the control group (Figure 2A,B). Administration of CCl_4_ to rats caused similar macroscopic changes, but the changes in liver morphology were even more pronounced, in particular, the surface of the liver was characterized by even greater granularity than that in the TAA group (Figure 2C). In the BDL group, the liver was enlarged, compacted, and had a smooth surface with a greatly expanded diameter of the ligature site of the common bile duct of 5 to 8 mm, which was filled with bile (Figure 2D). The livers of rats in the I/R group were characterized not only by enlargement and compaction of the tissue but also by the formation of numerous adhesions between the liver lobes, resulting in the formation of an atypical round shape (Figure 2E). After administration of TAA, CCl_4_ as well as modeling BDL, an increase in relative liver mass by 19%, 40%, and 38%, respectively, was observed, whereas liver mass of animals 6 weeks after exposure to I/R was comparable to the values of the control group (Figure 2F).

**Figure 3:**
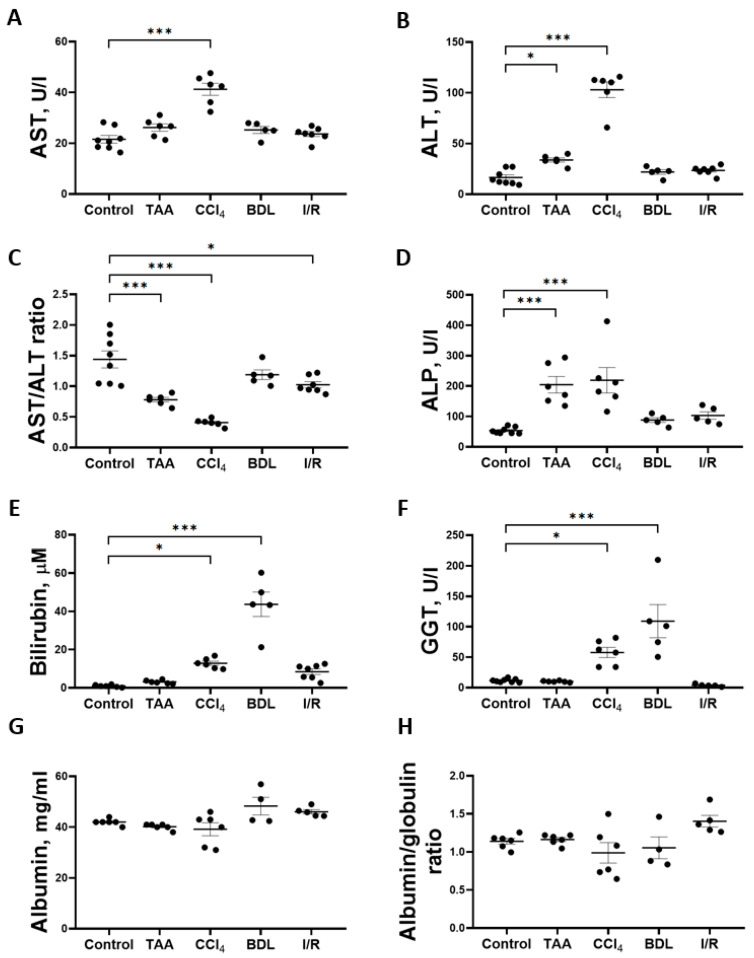
Development of liver damage in different fibrosis models. (**A**) Activity of AST in blood serum. (**B**) Activity of ALT in blood serum. (**C**) AST/ALT ratio in blood serum. (**D**) Activity of ALP in blood serum. (**E**) Bilirubin concentration in blood serum. (**F**) GGT activity in blood serum. (**G**) Albumin concentration in blood serum. (**H**) Albumin–globulin ratio in blood serum. The number of rats n ≥ 4 in all experimental groups. * *p* < 0.05, *** *p* < 0.001 (one-way ANOVA).

Determination of the activity of marker liver enzymes and the concentration of total bilirubin showed that the greatest changes were observed in the CCl_4_, TAA, and BDL groups (Figure 3). Chronic administration of CCl_4_ to rats resulted in a 2-fold increase in the activity of AST (Figure 3A) and a 6-fold increase in the activity of ALT (Figure 3B) and a 3.5-fold decrease in the AST/ALT ratio (Figure 3C). In the serum of rats receiving TAA, a 2-fold increase in the activity of ALT (Figure 3B) was observed with no change in the activity of AST, resulting in a 2.3-fold decrease in the AST/ALT ratio (Figure 3C). In rats exposed to I/R, there was only a moderate 29% decrease in the AST/ALT ratio (Figure 3C), with no significant increase in the absolute activity of ALT and AST, whereas the values of other liver indicators were close to those of the control group (Figure 3). Administration of CCl_4_ and TAA to rats was also accompanied by a 4-fold increase in ALP activity (Figure 3D). Thus, an increase in transaminase activity associated with a decrease in the AST/ALT ratio and an increase in ALP activity in blood serum may indicate the hepatotoxic effect of CCl_4_ and TAA. No changes in serum transaminase activities were detected in rats in the BDL group, but a significant 44-fold increase in total bilirubin concentration (Figure 3E) and 9.4-fold increase in GGT activity (Figure 3F) were typical of the development of obstructive cholestasis. A 12.8-fold increase in total bilirubin concentration in the serum of rats was also observed in the CCl_4_ group (Figure 3E), as well as GGT activity was also increased (Figure 3F). No hyperbilirubinemia developed in rats in the TAA and I/R groups (Figure 3E). The level of albumins and the albumin–globulin ratio in the serum of rats in all experimental groups did not decrease, indicating that the protein synthesis function of the liver was not impaired (Figure 3G,H).

**Figure 4:**
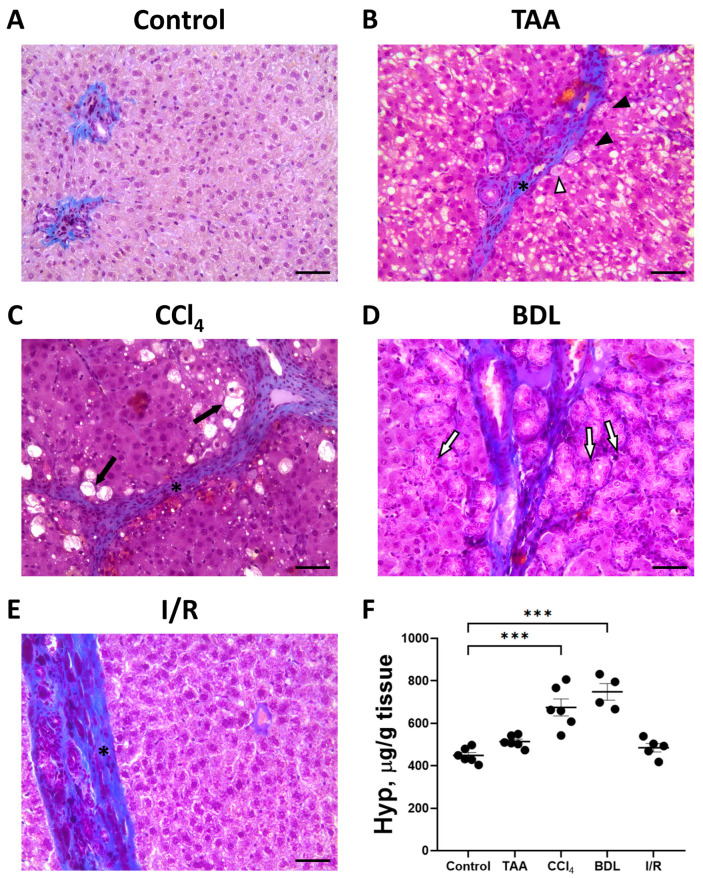
Development of liver fibrosis in different fibrosis models. Representative histological sections of the liver after Mallory staining. (**A**) Control rat. (**B**) 6 weeks after TAA administration. Hepatocytes with vacuoles (black arrowheads), hepatocytes with vacuoles and pyknotic nuclei (white arrowhead), and connective tissue septa (black asterisk) are observed. (**C**) 6 weeks after CCl_4_ administration. Dying hepatocytes (black arrows) and connective tissue septa (black asterisk) are observed. (**D**) 6 weeks after BDL with enlarged bile ducts (white arrows). (**E**) 6 weeks after I/R demonstrating connective tissue septa (black asterisk). Bar—100 μm. (**F**) Content of Hyp in the animal’s liver 6 weeks after modeling the injury. The number of rats n > 4 in all experimental groups. *** *p* < 0.001 (one-way ANOVA).

The development of liver fibrosis in rats was also evaluated on histological sections with Mallory staining (Figure 4A–E). In control animals, collagen fibers were mainly visible around the portal triads. In all experimental groups, there was some increase in blue-stained collagen fibers and morphologic changes characteristic of fibrosis.

When the liver was damaged with TAA, an increase in connective tissue around the portal vein was noticed with the formation of dense connective tissue septa around the lobules (Figure 4B). In addition to an increase in connective tissue, a characteristic feature of liver injury with TAA was a marked reaction of hepatocytes. Vacuoles of different sizes were observed in almost all hepatocytes, and some hepatocytes were characterized by pyknotic nuclei. Moderate proliferation of bile ducts was noted in some animals.

Chronic administration of CCl_4_ to experimental animals resulted in severe liver cirrhosis (Figure 4C). Severe changes in the liver histology was observed using sections stained with Mallory stain. Dense connective tissue septa separating the liver lobules became visible. Dying hepatocytes were located at the periphery of the liver lobules, which were characterized by pyknotic nuclei and complete destruction of the cytoplasm.

Liver injury with BDL revealed a significant increase in blue-stained collagen fibers, which were mainly located around the portal triads and along the bile ducts (Figure 4D). In addition, there was significant proliferation resulting in enlarged bile ducts and formation of large cystic cavities filled with bile in the portal areas (Figure 4D).

In the I/R group, liver tissue was characterized by an increase in blue-stained collagen fibers and an increase in the number of Kupffer cells, while the architecture of the liver lobules was largely preserved (Figure 4E).

### 3.2. Liver Fibrosis Is Accompanied by Changes in Glucose Metabolism

**Figure 5:**
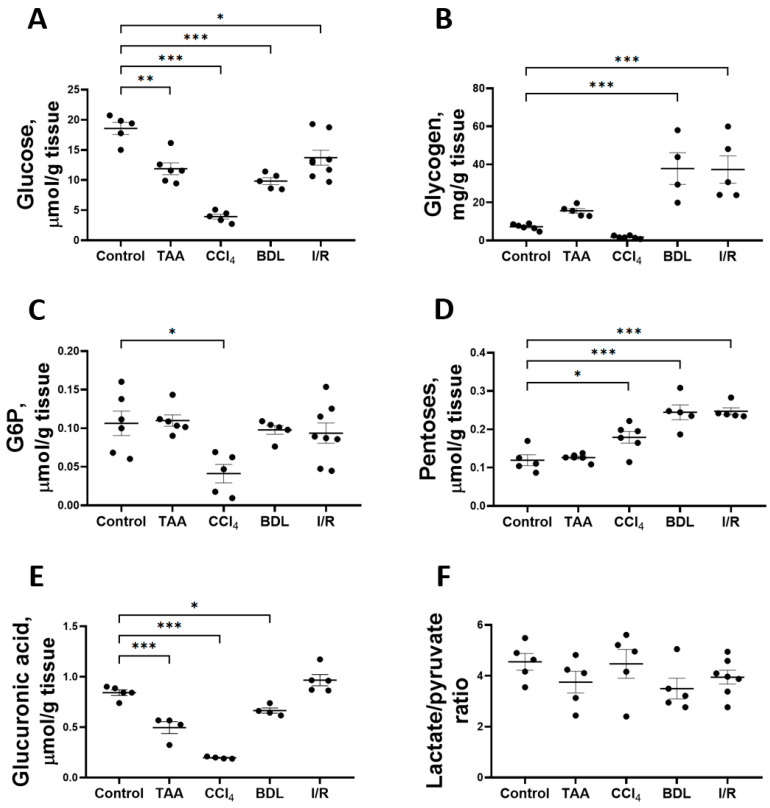
Level of glucose and its metabolites in rat liver tissue after damage modeling. (**A**) Glucose content. (**B**) Glycogen content. (**C**) Glucose-6-phosphate content. (**D**) Pentose content. (**E**) Glucuronic acid content. (**F**) Lactate/pyruvate ratio. The number of rats n ≥ 4 in all experimental groups. * *p* < 0.05, ** *p* < 0.01, *** *p* < 0.001 (one-way ANOVA).

Fibrotic changes formed as a result of prolonged administration of TAA and CCl_4_ to animals and in cholestatic and I/R liver injury resulted in the modulation of glucose metabolism—alterations in the content of substrates and activities of glycolytic enzymes, PPP, and the glucuronidation pathway (Figure 5). Thus, in all experimental models of liver injury, a significant decrease in glucose content in liver tissue was observed after 6 weeks (Figure 5A). In the animals of the CCl_4_ group, the glucose content in the liver decreased by 79%, whereas this indicator decreased by 36%, 47%, and 26% in the rats of the TAA, BDL, and I/R groups, respectively.

The induced changes in hepatic glucose levels were accompanied by changes in glycogen levels (Figure 5B). Liver glycogen levels were increased 5-fold in the BDL and I/R groups, whereas glycogen levels in the TAA and CCl_4_ groups were similar to those in the control group. Glucose-6-phosphate (G6P) content in the liver of rats decreased by 61% only when CCl_4_ was administered (Figure 5C). The decrease in glucose content in the liver was accompanied by an increase in the pentose content in all experimental groups except the TAA group (Figure 5D). On the other hand, glucuronic acid content decreased by 41%, 77%, and 21% in the TAA, CCl_4_, and BDL groups, respectively (Figure 5E). Despite the decrease in glucose content and the increase in pentose content in liver tissue, the lactate/pyruvate ratio remained unchanged in all experimental groups (Figure 5F). In addition, the concentrations of glucose, lactate, and pyruvate in serum were measured (Supplementary Figure S2). Administration of CCl_4_ decreased the blood glucose level and increased the pyruvate concentration (Supplementary Figure S2A,B). In other experimental groups, no changes were observed in these metabolites in blood or in lactate concentration (Supplementary Figure S2C). 

**Figure 6:**
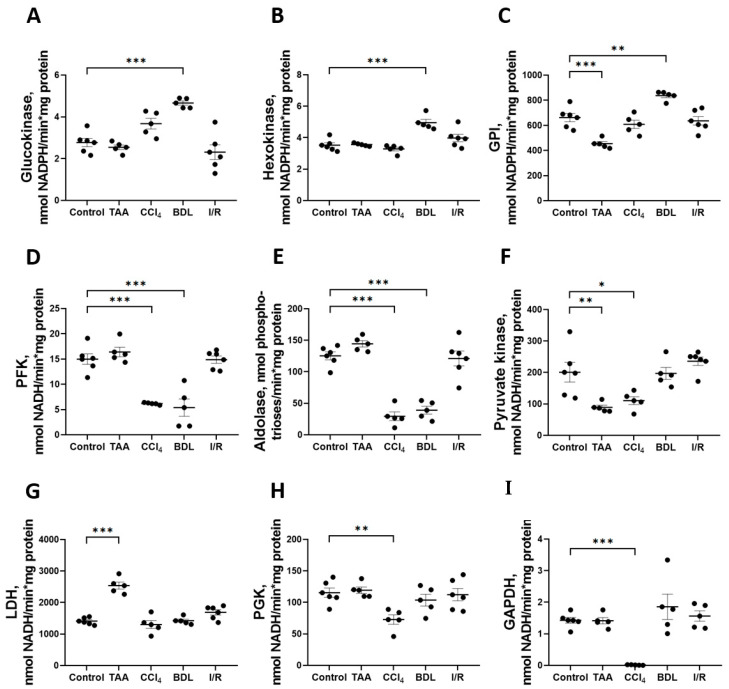
Changes in the activity of glycolytic enzymes in rat liver 6 weeks after injury. Glucokinase, hexokinase, glucose-6-phosphate isomerase, phosphofructokinase, aldolase, pyruvate kinase, lactate dehydrogenase, phosphoglycerate kinase, and glyceraldehyde-3-phosphate dehydrogenase activities ((**A**–**I**) correspondingly). The number of rats n ≥ 4 in all experimental groups. * *p* < 0.05, ** *p* < 0.01, *** *p* < 0.001 (one-way ANOVA).

The greatest changes in glycolytic enzyme activity were observed in the BDL, TAA, and CCl_4_ groups (Figure 6). In particular, 6 weeks after ligation of the common bile duct in rats, an increase in the activity of enzymes involved in the initial reactions of glycolysis was observed: GK activity increased by 68%, HK by 41%, and GPI by 26% (Figure 6A–C), whereas the activity of the rate-limiting glycolytic enzyme PFK decreased by 64% (Figure 6D) and fructose-1,6-bisphosphate aldolase by 69% (Figure 6E). No changes in the activity of the initial glycolytic enzymes were observed after 6 weeks of administration of TAA, but a decrease in GPI activity by 31% (Figure 6C) and PK by 56% (Figure 6F) was observed. It should be noted that only in the TAA group there was an increase in activity of the terminal glycolytic enzyme LDH (Figure 6G). Administration of CCl_4_ to rats for six weeks suppressed the activity of all glycolytic enzymes. In this group, a 59% decrease in PFK activity (Figure 6D), a 76% decrease in aldolase (Figure 6E), a 45% decrease in PK (Figure 6F), a 99% decrease in PGK (Figure 6H), and a dramatic 99% decrease in GAPDH activity (Figure 6I) were observed. No changes in glycolytic enzyme activities were observed in the I/R group (Figure 6). Additionally, we found that the GAPDH expression in liver tissue significantly changed during fibrosis induction (Supplementary Figure S3).

### 3.3. Activity of the Glucuronic Acid and Pentose Phosphate Pathways of Glucose Metabolism

**Figure 7:**
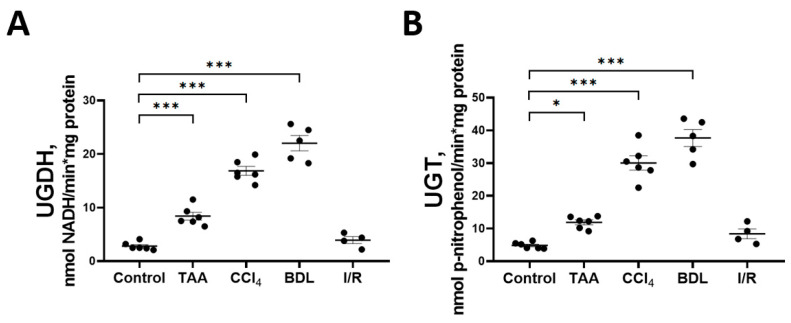
Changes in the activity of enzymes of the glucuronic pathway. (**A**) UDP-glucose dehydrogenase activity; (**B**) UDP-glucuronosyltransferase activity. The number of rats n ≥ 4 in all experimental groups. * *p* < 0.05, *** *p* < 0.001 (one-way ANOVA).

Decreases in the glucose and glucuronic acid content in the rat liver tissue in experimental fibrosis models were associated with activation of key enzymes of the glucuronic acid pathway UGDH and UGT (Figure 7). A more than 6-fold increase in the activity of UGDH and UGT was observed in the liver of rats exposed to BDL and CCl_4_. A significant increase in the activity of both enzymes was also observed in the liver of rats in the TAA group. No changes in the activity of UGDH and UGT were detected in the liver of rats from the I/R group. In addition, we found that the mRNA expression of the *UGT1A3* isoform was increased in the liver tissue of rats of the CCl_4_ group, whereas in the other groups there was a tendency for an increased expression of both the *UGDH* and *UGT1A3* isoforms as compared with the control group (Supplementary Figure S4).

**Figure 8:**
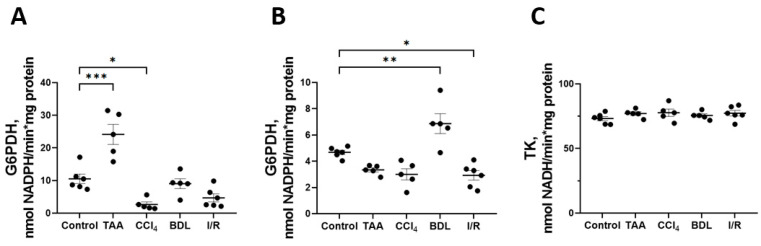
Changes in PPP enzyme activity in the rat liver tissue upon various injuries. (**A**) Glucose-6-phosphate dehydrogenase activity. (**B**) 6-phosphogluconate dehydrogenase activity. (**C**) Transketolase activity. The number of rats n ≥ 4 in all experimental groups. * *p* < 0.05, ** *p* < 0.01, *** *p* < 0.001 (one-way ANOVA).

Some changes were also observed in the activity of enzymes of the oxidative stage of PPP (Figure 8). In particular, a 2-fold increase in G6PDH activity was observed in the TAA group and a 4-fold decrease in its activity in the CCl_4_ group, *p* < 0.05 (Figure 8A). At the same time, the activity of 6PGDH in rat liver increased by 47% in the BDL group and decreased by 38% in the I/R group (Figure 8B). No changes in transketolase activity (TK) were detected in the livers of rats in all experimental groups (Figure 8C).

### 3.4. Liver Fibrosis Is Associated with Increased Activity of Krebs Cycle Enzymes and Impaired Respiratory Activity of Mitochondria

**Figure 9:**
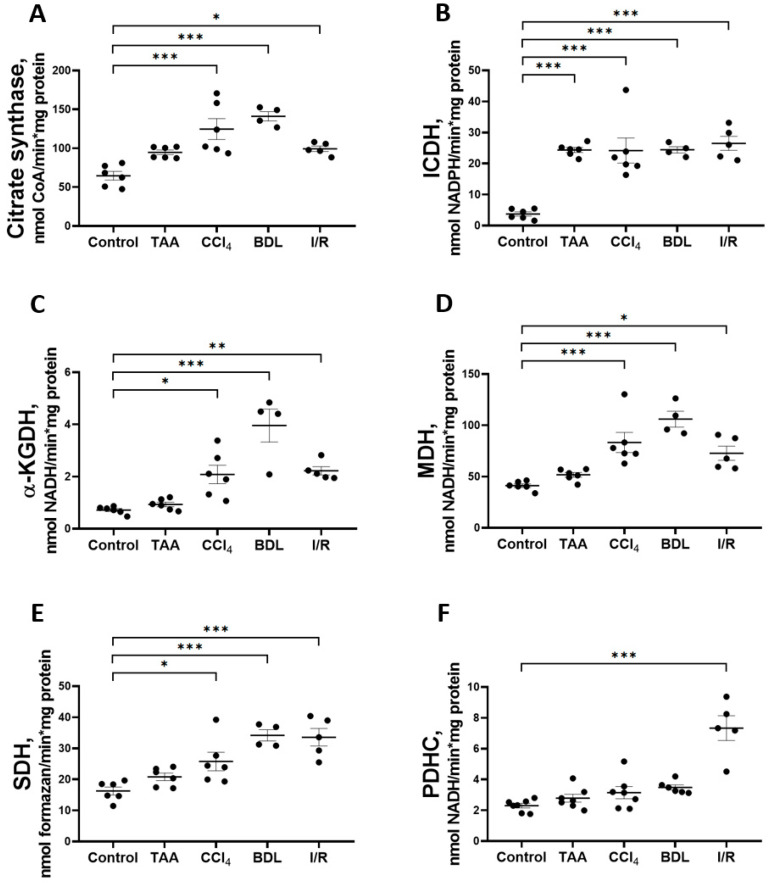
Changes in the activity of Krebs cycle enzymes and pyruvate dehydrogenase complex in rat liver tissue. (**A**) Citrate synthase activity. (**B**) Isocitrate dehydrogenase activity. (**C**) α-ketoglutarate dehydrogenase activity. (**D**) Malate dehydrogenase activity. (**E**) Succinate dehydrogenase activity. (**F**) Pyruvate dehydrogenase complex activity. The number of rats n ≥ 4 in all experimental groups. * *p* < 0.05, ** *p* < 0.01, *** *p* < 0.001 (one-way ANOVA).

**Figure 10:**
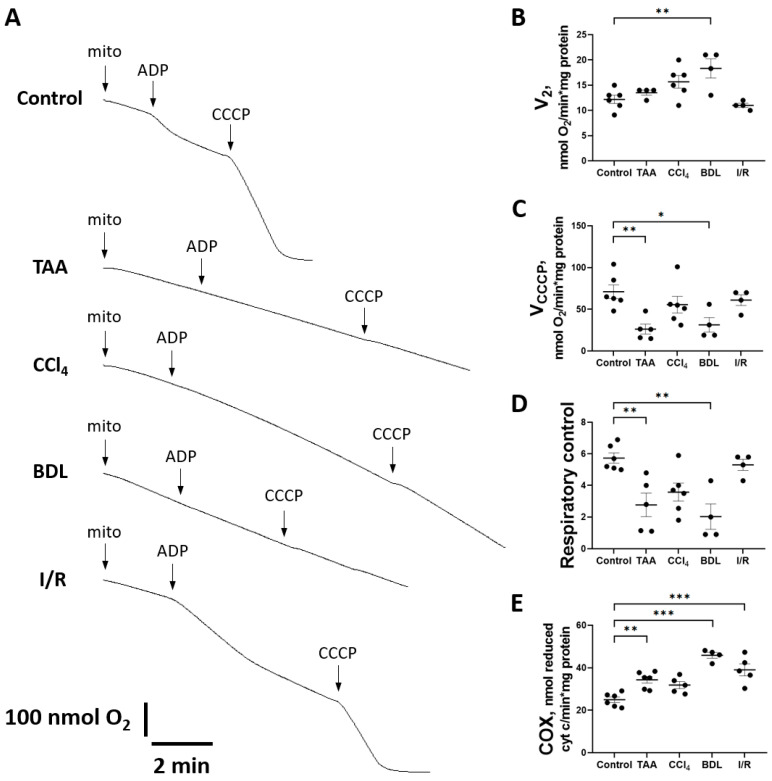
Changes in liver mitochondrial oxygen consumption during injury modeling. (**A**) Example polarographic curves showing basal respiration in state 2 and respiration after ADP and CCCP supplementation (state 3). (**B**) Estimation of state 2 respiration rate. (**C**) Estimation of respiration rate after CCCP addition. (**D**) Respiratory control measured as V_CCCP_/V_2_ ratio. (**E**) Cytochrome c oxidase activity. The number of rats n ≥ 4 in all experimental groups. * *p* < 0.05, ** *p* < 0.01, *** *p* < 0.001 (one-way ANOVA).

In addition to studying the major metabolic pathways of glucose conversion in the rat liver fibrosis models, the activity of Krebs cycle enzymes (Figure 9) was measured as well as the parameters of mitochondrial respiratory function (Figure 10). The activity of CS in liver mitochondria increased to 193%, 218%, and 154% after administration of CCl_4_, modeling BDL, and I/R, respectively, compared to the control group (Figure 9A). These groups also showed a 6-fold increase in the activity of the rate-limiting enzyme of the Krebs cycle, ICDH (Figure 9B), and a more than 2-fold increase in the activity of α-KGDH (Figure 9C), MDH (Figure 9D), and SDH (Figure 9E). In addition, a 3-fold increase in PDHC activity was detected in the I/R group, whereas it did not change in the other experimental groups (Figure 7F). In the rats receiving TAA, all these changes were less pronounced than in the other groups, and only a 6-fold increase in ICDH activity was significant (Figure 7B). 

When mitochondrial respiration rate was measured with succinate as substrate (Figure 10A), no significant differences were observed in state V_2_, except in the BDL group (Figure 10B). However, a significant decrease in the rate of uncoupled mitochondrial respiration V_CCCP_ (state 3u after addition of CCCP) was observed in all experimental groups except I/R (Figure 10C). However, during treatment with TAA and modeling of BDL, respiratory control was significantly reduced (Figure 10D).

It should be noted that in animals with liver fibrosis, an increase in the activity of the complexes II and IV occurred simultaneously with signs of mitochondrial respiratory dysfunction. In addition to the aforementioned increase in SDH activity, a significant increase in cytochrome c oxidase (COX) activity was observed in the BDL and I/R, TAA, and CCl_4_ groups (Figure 10E).

### 3.5. Liver Fibrosis Was Accompanied by a Decrease in Antioxidant Activity and the Development of Oxidative Stress

**Figure 11:**
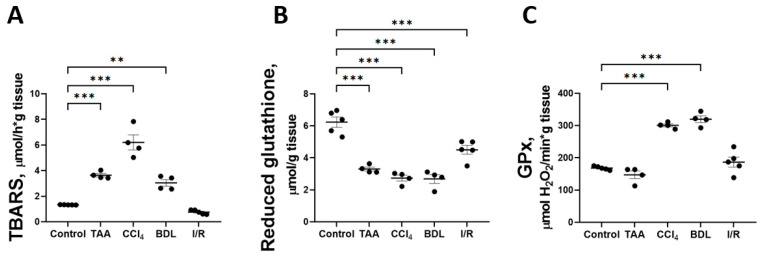
Parameters of lipid peroxidation and antioxidant system in liver tissue against various injuries. (**A**) TBARS level induced by Fe^2+^/ascorbate. (**B**) Reduced glutathione content. (**C**) Activity of glutathione peroxidase. The number of rats n ≥ 4 in all experimental groups. ** *p* < 0.01, *** *p* < 0.001 (one-way ANOVA).

One explanation for the disruption of glucose metabolism and impaired respiratory chain function could be the oxidation by reactive oxygen species (ROS) of reactive sulfhydryl groups and FeS clusters of proteins that are part of the multienzyme complexes of the mitochondrial electron transport chain. Accordingly, the indicators of oxidative stress in liver tissue were analyzed in all fibrosis models (Figure 11). It was found that induction of in vitro Fe^2+^/ascorbate lipid peroxidation was accompanied by an increase in the formation of carbonyl compounds reactive with 2-thiobarbituric acid (TBARS) in all liver injury models compared with the control (Figure 11A). The Fe^2+^/ascorbate-induced TBARS level was 4.6-fold higher in the CCl_4_ group and 2-fold higher in the TAA and BDL groups than in the control group. This indicates a decrease in antioxidative potential in liver tissue, probably due to the depletion of redox buffer compounds under the conditions of oxidative stress induced by pathology. Indeed, a significant decrease in the content of reduced glutathione in liver tissue was observed in all fibrosis models compared with the control group (Figure 11B). In addition, the activity of the antioxidant enzyme glutathione peroxidase (GPx) was found to be significantly increased in the rat liver tissue after CCl_4_ administration and modeling from BDL, whereas GPx activity remained unchanged in the TAA and I/R groups (Figure 11C).

### 3.6. Bioinformatic Analysis of the Metabolic Profile of the Liver

**Figure 12:**
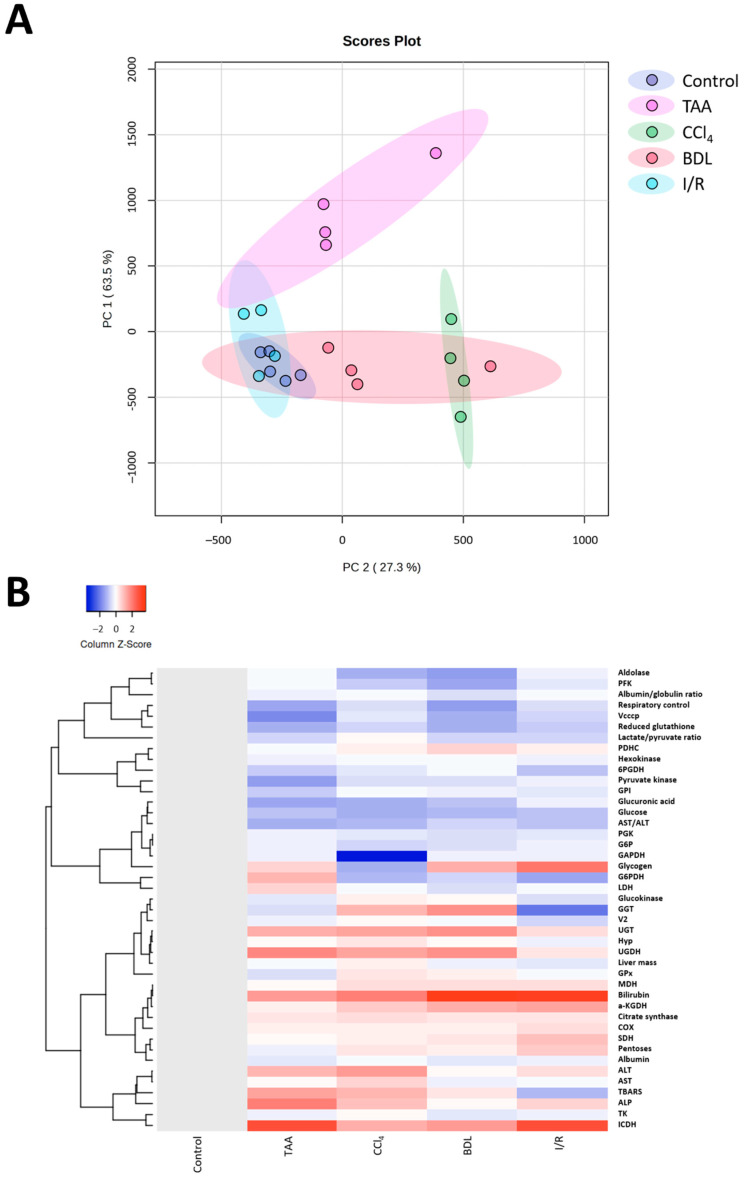
Influence of different injuries on the metabolic profile of the liver. (**A**) PC analysis of metabolic changes in blood and liver tissue after different models of liver injury. (**B**) Clustering analysis of metabolic changes in blood and liver tissue after different models of liver injury.

Multivariate analysis of the obtained results using principal component analysis (PC) showed differences in the metabolic profile of rats after performing different fibrosis models (Figure 12A). Rats in the control group were characterized by low parameter variability, in contrast to rats with liver damage. Clear clustering and differentiation from the control group was observed for the TAA, CCl_4_, and BDL groups, whereas the I/R group partially overlapped with the control group. It was observed that the CCl_4_ and BDL groups had significant similarities in metabolic profile, while the TAA group, although different from the control group, was not clustered with the other groups. This suggested that clustering based on metabolic changes reflected changes in the degree of fibrosis, indicating that fibrosis was associated with the greatest metabolic shifts. In addition, cluster analysis of all measured parameters in the study was performed, and the results confirmed the data from the PC analysis that liver fibrosis significantly altered the metabolic profile of cells (Figure 12B).

## 4. Discussion

In this study, we evaluated the severity of liver damage and morphological changes six weeks after induction of four of the most well-known models of liver pathologies. Concentrations of glucose metabolites, as well as the activity of glycolysis and tricarboxylic acid cycle (TCA) enzymes, were analyzed in liver tissue. Moreover, the respiratory activity of liver mitochondria, the activity of PPP enzymes, and the level of oxidative stress were studied. Similarities and differences in the formation of fibrotic scar in different types of liver damage were investigated, as well as their correlation with changes in bioenergetics and glucose metabolism.

Long-term administration of TAA and CCl_4_ to animals and modeling of cholestatic and ischemic/reperfusion liver damage led to CLD, as confirmed by morphological changes, increased activity of serum liver enzymes (in all experimental groups), and increased concentration of total bilirubin (in CCl_4_ administration and BDL modeling). These changes were accompanied by a significant increase in Hyp content in the rat livers of the CCl_4_ and BDL groups, which was the index of the fibrosis progression. It should be noted that the degree of fibrosis was different in all models of liver injury, indicating some differences in the pathogenesis of these pathologies. It has been previously shown that the severity of fibrosis may be related to the doses and administration protocol of CCl_4_ and TAA [35], the duration of ligation of the common bile duct [36], or the duration of ischemia and reperfusion [37,38]. Prolonged administration of CCl_4_ and TAA for 6–8 weeks has been used to model liver fibrosis in rats and mice [36], while prolonged exposure (10–12 weeks) to these compounds has been used to model liver cirrhosis [39]. BDL leads to maximal fibrotic changes in the liver of rats after 6 weeks [40], whereas in the I/R modeling, the most pronounced fibrotic changes in the liver were observed after 4–7 days and attenuated somewhat at 2 weeks after the intervention [38]. In our experiment, there was no increase in the activity of marker liver enzymes up to 6 weeks in the serum of rats whose liver was exposed to I/R, which was probably due to the high regenerative capacity of liver cells that compensate a relatively short damaging effect [41]. The severity of fibrotic changes in the I/R model was also small, although morphological changes in the liver (increase in size and tissue density, formation of adhesions between lobes) persist in animals after 6 weeks of I/R, as do deposits of collagen fibers in histochemical staining, although in lower expression than in other models. 

The liver is the main organ responsible for detoxification of xenobiotics and it plays a central role in the metabolic homeostasis of the organism by providing synthesis, conversion, storage and redistribution of carbohydrates, lipids and their metabolites between tissues [42]. In our study, a significant decrease in glucose content was observed in the TAA, CCl_4_, BDL and I/R groups, while glucose-6-phosphate content and blood glucose level decreased significantly in the liver of rats only in the CCl_4_ group. In the other groups (TAA, BDL, and I/R), the glucose-6-phosphate content in the liver and the blood glucose level were not distinguished from the control values. Liver glycogen content decreased significantly in animals in the TAA and CCl_4_ groups, probably due to the high demand of liver cells for glucose to synthesize UDP-glucuronate, which was required for glucuronidation reactions to detoxify these xenobiotics. In the BDL and I/R groups, there was a significant increase in glycogen levels in rat liver tissue. This may be due to the accumulation of bile acids in BDL and the inhibition of cAMP formation by a mechanism involving protein kinase C, probably leading to a modulation of glycogen metabolism in the liver [43]. It should be noted that the collection of liver tissue in all experimental groups was performed after 3 h of fasting, so that the level of blood glucose and the content of glucose and its exchange products were clearly comparable in all experimental groups. For other elements of carbohydrate metabolism, we also found no changes in the I/R group, which was consistent with the low development of fibrosis and was probably due to the high regenerative capacity of the post-ischemic damaged liver [38,41].

We revealed significant changes in the activity of glycolytic enzymes in rat liver in the BDL, CCl_4_, TAA, and I/R groups, and the nature of these changes varied among the experimental groups. It should be noted that the specific activity of the enzymes was studied at the initial reaction rates with the excess of substrate, so that the activity apparently depended on the amount of enzyme in the samples rather than on allosteric or substrate inhibition. We attributed such changes in the activity of the enzymes mainly to a change in their expression or inhibition by damage. In the BDL group, there was a change in the activity of the initial glycolysis enzymes (GK, HK, and GPI) and a decrease in the activity of PFK and aldolase. In animals in the CCl_4_ and TAA groups, no changes in the activity of upstream glycolytic enzymes were observed in liver tissue, whereas in rat liver in the CCl_4_ group, the most dramatic changes in the activity of enzymes catalyzing downstream steps, especially a marked decrease in aldolase and almost complete inhibition of GAPDH activity, were observed. It has been known that GAPDH is one of the most susceptible target proteins in oxidative stress [44], and probably that GAPDH undergoes post-translational oxidative modification (S-thiolation) and inactivation upon induction of oxidative stress by CCl_4_ [45,46]. However, in our study, we did not specifically consider the post-translational modifications that may also regulate enzyme activity. Another feature of the changes in glycolysis in the CCl_4_ group was the suppression of the activity of kinase enzymes (PFK, PGK, and PK). It should be noted that no changes in the activity of glycolytic enzymes were detected in the I/R group, which was consistent with a weak change in the level of glycolysis metabolites. 

Metabolic changes are crucial for the development of fibrotic changes [47]. The production of compounds that are structural components of the extracellular matrix is an energy-consuming process, and glycolysis is one of the predominant metabolic pathways in fibroblasts of various organs, including the liver [48]. Increased expression of glycolytic enzymes in fibroblasts during fibrosis has been demonstrated in several studies. For example, differentiation of lung fibroblasts is associated with high glucose consumption and lactate production and depends on the expression of phosphofructokinase/fructose-2,6-bisphosphatase (PFKFB3), which converts fructose-6-phosphate to fructose-2,6-bisphosphate, a potent allosteric activator of PFK [49]. A significant increase in lactate production was also observed in a model of renal interstitial fibrosis in mice, which was associated with increased expression of HK and PK [49,50,51]. We also observed changes in the activity of glycolytic enzymes in models of liver injury characterized by the most severe development of fibrosis. Thus, the changes in the activity of glycolytic enzymes that we found may be potential molecular targets for the regulation not only of energy processes but also of fibrogenesis processes.

Along with changes in enzymes of glycolysis, activation of enzymes of the glucuronate pathway and PPP was also observed. PPP is an alternative pathway for the oxidation of glucose-6-phosphate in hepatocytes, providing the cell with pentoses and NADPH, which are necessary for many processes including functioning of the glutathione redox system and biosynthetic processes (such as the synthesis of fatty acids and cholesterol) [52,53]. In the glucuronate pathway, UDP-glucuronate is formed, which provides reactions for the conjugation of bilirubin and various xenobiotics and is a key pathway for their detoxification and excretion from the organism [54]. Indeed, together with a decrease in glucose content in the liver of rats in the groups TAA, CCl_4_, BDL and I/R, we observed an increase in pentoses content. The main supplier of pentoses in the liver is PPP, but only in the groups TAA and BDL an increase in the activity of the initial PPP enzymes—G6PDH and 6PGDH—was observed. On the other hand, we found a significant increase in UGDH and UGT activity in all experimental groups except I/R, indicating the great importance of the glucuronate pathway in liver fibrosis in toxic and cholestatic damage. The highest levels of UGDH and UGT were detected in animals in the CCl_4_ and TAA groups, which occurred to be hyperbilirubinemic. We also found a significant decrease in the content of glucuronic acid in the liver tissue of animals in the groups TAA, CCl_4_, and BDL, which was probably related to the active use of UDP-glucose for conjugation reactions catalyzed by UGT. Thus, toxic liver injury activated the glucuronate pathway of the glucose metabolism, which required a substantial flux of glucose to be utilized, while the glucose flux through glycolysis was suppressed. Additionally, some glucose is apparently used to replenish the pentose pool, which, in addition to glycolysis and PPP, is used for the biosynthesis of ascorbic acid [55], which is a cofactor of prolyl and lysyl hydroxylase, which hydroxylates and forms mature collagen [56,57]. Ultimately, it yields redirection of the glucose flux in the liver metabolism under toxic environment.

As for the liver, activated HSCs have been shown to exhibit enhanced gluconeogenesis, expression of glucose transporters, and activation of glycolytic enzymes, which are also accompanied by changes in amino acid and fatty acid metabolism [58]. In addition to glycolysis, mitochondrial oxidative phosphorylation is activated in HSCs [59]. We demonstrated that together with a decrease in the activity of glycolytic enzymes in the liver of rats in the CCl_4_, BDL, and TAA groups, the activation of the Krebs cycle enzymes and suppression of mitochondrial respiratory activity occured. We revealed that V_2_ was unchanged in all groups except BDL, which may indicate the preserved barrier function of the inner mitochondrial membrane (Figure 10A,B). The decrease in maximal respiratory flux measured by uncoupled mitochondrial respiration rate (V_CCCP_) may indicate a disturbance in the functioning of the respiratory chain (Figure 10C). Since this decrease was observed with succinate as a substrate, this could indicate a retarded electron flow through the II–IV complexes of the respiratory chain. However, we did not observe a decrease in the activity of succinate dehydrogenase (Figure 9E) in these models, implying that the complex III and/or cytochrome c oxidase were primarily impaired possibly through retarded transfer between respiratory complexes and final electron acceptor, oxygen. Since in state 3u oxygen consumption rate was limited only by the electron flow and substrate transport into mitochondria, these two options could be considered (e.g., inhibition of a dicarboxylate transporter, cytochrome c release or cytochrome oxidase inhibition by intrinsic inhibitors such as NO). Moreover, we could not exclude that in these conditions, the mitochondria were fully uncoupled. Indeed, during treatment with TAA and modeling of BDL, respiratory control was significantly reduced (Figure 10D). It was likely that the Krebs cycle activation was related to the uptake of alpha-keto acids and amino acids, which were actively formed in the liver by transamination reactions. Another possible mechanism for the activation of TCA cycle could be mediated by short-chain acyl-CoA, which was actively formed in the liver as a result of the beta-oxidation of fatty acids.

The suppression of mitochondrial respiratory function and the decrease in the activity of liver glycolytic enzymes in fibrosis of rats caused by the four agents studied might be associated with oxidative stress [60,61]. In TAA and CCl_4_, alkyl halogenated radicals and oxidation products of TAA (thioacetamide S-oxide) and ROS generated during their neutralization in the liver are sources of this stress [62,63]. Dysfunction of the multifunctional complexes of the respiratory chain of mitochondria may be due to the oxidation of iron–sulfur clusters under the action of ROS, resulting in disturbances of oxidative phosphorylation [64]. In our experiment, disturbances in the balance between prooxidants and antioxidants in the liver were revealed by an increased sensitivity to the induction of lipid peroxidation by Fe^2+^/ascorbate and a significant reduction in reduced glutathione. Thus, we could conclude that in the development of CLD, the maintenance of the redox balance was a crucial factor that ensures the normal function of mitochondria and the flow of cellular metabolism, which contributed to the overcoming of fibrotic changes in liver tissue.

The development of a targeted therapy for liver fibrosis is of great clinical importance. Since significant metabolic changes have been observed in liver cells after exposure to damaging factors, targeted treatment could be based on metabolic modulators. HSCs seem to be one of the preferred targets, because these cells, which are responsible for the formation of fibrotic tissue in the liver, are characterized by the high expression and activity of glycolytic enzymes [65]. Several glycolysis inhibitors and hypoglycemic agents have been recently studied and showed promising results for the treatment of liver fibrosis in vitro and in vivo models [66,67,68,69]. The results of our work were another step in this direction.

## 5. Limitations of the Study

In our study, we examined the long-term effects of chronic administration of TAA, CCl_4_ or modeling cholestatic and I/R injury on liver tissue. For all models, we chose a period of 6 weeks to study morphological or metabolic changes associated with the development of liver fibrosis. However, given the large regenerative capacity of the liver, shorter periods may be required to detect more pronounced metabolic changes in acute injuries such as liver I/R. This study was performed only on males and did not consider the effects of sex on the metabolic changes observed during the development of liver fibrosis in different models.

## 6. Conclusions

**Figure 13:**
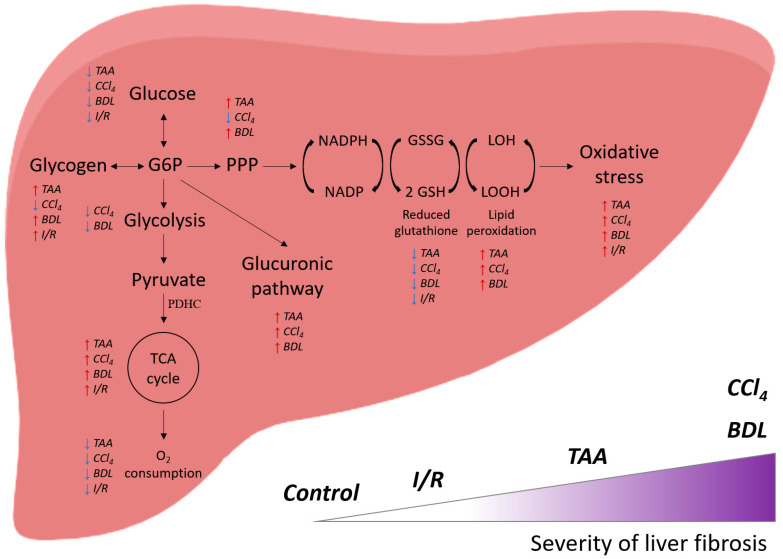
General scheme of the effect of various damage models on the energy metabolism of the liver and their correlation with the severity of fibrosis.

Thus, administration of CCl_4_, TAA to animals and modeling BDL (and to a lesser extent I/R) led to the development of liver damage and fibrosis, the extent of which correlates with changes in glucose metabolism: suppression of glycolysis, activation of the glucuronidation pathway and PPP, and accompanied by significant activation of the Krebs cycle enzymes with concomitant suppression of mitochondrial respiration (Figure 13). These changes were accompanied by a decrease in liver antioxidant status and a decrease in glutathione levels in a background of activation of lipid peroxidation. It could be concluded that the extent of impairment of energy metabolism in the liver depended on the pathology, and its association with fibrosis was more characteristic of BDL and the effects of CCl_4_ and less of I/R, which should be taken into account when developing therapeutic strategies.

## Figures and Tables

**Table 1:** Primer sequences used for estimation of gene expression.

Gene Name	*The Sequence of Forward Primer 5′- to 3′*	*The Sequence of Reverse Primer 5′- to 3′*
*UGDH*	AGCCATCAAGGACCTAAAGAACCC	TTGCCACCTCTTCCACATCGG
*UGT1A3*	TCTGGACCTGGCTGTGTTCTG	AGACAATGAAGACCACCGTCAAC

## Data Availability

Data generated and analyzed during the current study are available from the corresponding author on reasonable request.

## Supplementary Materials

The following supporting information can be downloaded at: https://www.mdpi.com/article/10.3390/antiox12081604/s1, Table S1: The number of rats in each experimental group; Figure S1: Liver injury markers in control groups; Figure S2: Glucose and its metabolites in the whole blood of rats with various liver lesions; Figure S3: GAPDH content in rat liver tissue with various injuries; Figure S4: Expression of enzymes of the glucuronidation pathway.

## References

[1] Asrani S.K., Devarbhavi H., Eaton J., Kamath P.S. (2019). Burden of Liver Diseases in the World. J. Hepatol..

[2] Roehlen N., Crouchet E., Baumert T.F. (2020). Liver Fibrosis: Mechanistic Concepts and Therapeutic Perspectives. Cells.

[3] Parola M., Pinzani M. (2019). Liver Fibrosis: Pathophysiology, Pathogenetic Targets and Clinical Issues. Mol. Aspects Med..

[4] Kisseleva T., Brenner D. (2021). Molecular and Cellular Mechanisms of Liver Fibrosis and Its Regression. Nat. Rev. Gastroenterol. Hepatol..

[5] Caligiuri A., Gentilini A., Pastore M., Gitto S., Marra F. (2021). Cellular and Molecular Mechanisms Underlying Liver Fibrosis Regression. Cells.

[6] Ung C.Y., Onoufriadis A., Parsons M., McGrath J.A., Shaw T.J. (2021). Metabolic Perturbations in Fibrosis Disease. Int. J. Biochem. Cell Biol..

[7] Yakupova E.I., Zorov D.B., Plotnikov E.Y. (2021). Bioenergetics of the Fibrosis. Biochemistry.

[8] Reddy G.K., Enwemeka C.S. (1996). A Simplified Method for the Analysis of Hydroxyproline in Biological Tissues. Clin. Biochem..

[9] Bücher T., Czok R., Lamprecht W., Latzko E. (1965). Pyruvate. Methods of Enzymatic Analysis.

[10] Zhu A., Romero R., Petty H.R. (2011). An Enzymatic Colorimetric Assay for Glucose-6-Phosphate. Anal. Biochem..

[11] Mckay E. (1964). Pentose Estimation by the Orcinol Method, with Particular Reference to Plasma Pentose. Clin. Chim. Acta.

[12] Tomasić J., Keglević D. (1972). Direct Spectrophotometric Assay of Glucuronic Acid in the Presence of Labile Glucosiduronic Acids. Anal. Biochem..

[13] Bennett L.W., Keirs R.W., Peebles E.D., Gerard P.D. (2007). Methodologies of Tissue Preservation and Analysis of the Glycogen Content of the Broiler Chick Liver. Poult. Sci..

[14] Pilkis S.J. (1975). Glucokinase of Rat Liver. Methods Enzymol..

[15] Gracy R.W. (1982). Glucosephosphate Isomerase from Catfish Muscle and Liver and from Mammalian Tissues. Methods Enzymol..

[16] Harris J.I., Waters M., Boyer P.D. (1976). 1 Glyceraldehyde-3-Phosphate Dehydrogenase. The Enzymes.

[17] Kemp R.G. (1975). Phosphofructokinase from Rabbit Skeletal Muscle. Methods Enzymol..

[18] Krietsch W.K., Bücher T. (1970). 3-Phosphoglycerate Kinase from Rabbit Sceletal Muscle and Yeast. Eur. J. Biochem..

[19] Cardenas J.M. (1982). Pyruvate Kinase from Bovine Muscle and Liver. Methods Enzymol..

[20] Lee C.Y., Yuan J.H., Goldberg E. (1982). Lactate Dehydrogenase Isozymes from Mouse. Methods Enzymol..

[21] Dror Y., Sassoon H.F., Watson J.J., Johnson B.C. (1970). Glucose 6-Phosphate Dehydrogenase Assay in Liver and Blood. Clin. Chim. Acta.

[22] Hecquet L., Bolte J., Demuynck C. (1993). New Assays for Transketolase. Biosci. Biotechnol. Biochem..

[23] Campbell R.E., Sala R.F., van de Rijn I., Tanner M.E. (1997). Properties and Kinetic Analysis of UDP-Glucose Dehydrogenase from Group A Streptococci. Irreversible Inhibition by UDP-Chloroacetol. J. Biol. Chem..

[24] Puukka R., Tanner P., Hänninen O. (1973). Enzymes of the Glucuronic Acid Pathway in Wistar and Gunn Rats and Their Heterozygotes. Biochem. Genet..

[25] Sibley J.A., Lehninger A.L. (1949). Determination of Aldolase in Animal Tissues. J. Biol. Chem..

[26] Hinman L.M., Blass J.P. (1981). An NADH-Linked Spectrophotometric Assay for Pyruvate Dehydrogenase Complex in Crude Tissue Homogenates. J. Biol. Chem..

[27] Spinazzi M., Casarin A., Pertegato V., Salviati L., Angelini C. (2012). Assessment of Mitochondrial Respiratory Chain Enzymatic Activities on Tissues and Cultured Cells. Nat. Protoc..

[28] Bergmeyer H.-U. (1974). Isocitrate Dehydrogenase. Methods of Enzymatic Analysis.

[29] Semenovich D.S., Plotnikov E.Y., Titko O.V., Lukiyenko E.P., Kanunnikova N.P. (2021). Effects of Panthenol and N-Acetylcysteine on Changes in the Redox State of Brain Mitochondria under Oxidative Stress In Vitro. Antioxidants.

[30] Green J.D., Narahara H.T. (1980). Assay of Succinate Dehydrogenase Activity by the Tetrazolium Method: Evaluation of an Improved Technique in Skeletal Muscle Fractions. J. Histochem. Cytochem..

[31] Bisswanger H. (2011). Practical Enzymology: BISSWANGER:PRACT.ENZYM.E2 O-BK.

[32] Wei Q.-Y., Chen W.-F., Zhou B., Yang L., Liu Z.-L. (2006). Inhibition of Lipid Peroxidation and Protein Oxidation in Rat Liver Mitochondria by Curcumin and Its Analogues. Biochim. Biophys. Acta.

[33] Patsoukis N., Georgiou C.D. (2004). Determination of the Thiol Redox State of Organisms: New Oxidative Stress Indicators. Anal. Bioanal. Chem..

[34] Flohé L., Günzler W.A. (1984). Assays of Glutathione Peroxidase. Methods Enzymol..

[35] Yanguas S.C., Cogliati B., Willebrords J., Maes M., Colle I., van den Bossche B., de Oliveira C.P.M.S., Andraus W., Alves V.A.F., Leclercq I. (2016). Experimental Models of Liver Fibrosis. Arch. Toxicol..

[36] Abshagen K., König M., Hoppe A., Müller I., Ebert M., Weng H., Holzhütter H.-G., Zanger U.M., Bode J., Vollmar B. (2015). Pathobiochemical Signatures of Cholestatic Liver Disease in Bile Duct Ligated Mice. BMC Syst. Biol..

[37] Olthof P.B., van Golen R.F., Meijer B., van Beek A.A., Bennink R.J., Verheij J., van Gulik T.M., Heger M. (2017). Warm Ischemia Time-Dependent Variation in Liver Damage, Inflammation, and Function in Hepatic Ischemia/reperfusion Injury. Biochim. Biophys. Acta Mol. Basis Dis..

[38] Konishi T., Schuster R.M., Lentsch A.B. (2019). Liver Repair and Regeneration after Ischemia-Reperfusion Injury Is Associated with Prolonged Fibrosis. Am. J. Physiol. Gastrointest. Liver Physiol..

[39] Nevzorova Y.A., Boyer-Diaz Z., Cubero F.J., Gracia-Sancho J. (2020). Animal Models for Liver Disease—A Practical Approach for Translational Research. J. Hepatol..

[40] Garrido M., Escobar C., Zamora C., Rejas C., Varas J., Párraga M., San Martin S., Montedónico S. (2017). Bile Duct Ligature in Young Rats: A Revisited Animal Model for Biliary Atresia. Eur. J. Histochem..

[41] Konishi T., Schuster R.M., Goetzman H.S., Caldwell C.C., Lentsch A.B. (2020). Fibrotic Liver Has Prompt Recovery after Ischemia-Reperfusion Injury. Am. J. Physiol. Gastrointest. Liver Physiol..

[42] Raddatz D., Ramadori G. (2007). Carbohydrate Metabolism and the Liver: Actual Aspects from Physiology and Disease. Z. Gastroenterol..

[43] Lang C., Schäfer M., Varga L., Zimmermann A., Krähenbühl S., Krähenbühl L. (2002). Hepatic and Skeletal Muscle Glycogen Metabolism in Rats with Short-Term Cholestasis. J. Hepatol..

[44] Hwang N.R., Yim S.-H., Kim Y.M., Jeong J., Song E.J., Lee Y., Lee J.H., Choi S., Lee K.-J. (2009). Oxidative Modifications of Glyceraldehyde-3-Phosphate Dehydrogenase Play a Key Role in Its Multiple Cellular Functions. Biochem. J..

[45] Grant C.M., Quinn K.A., Dawes I.W. (1999). Differential Protein S-Thiolation of Glyceraldehyde-3-Phosphate Dehydrogenase Isoenzymes Influences Sensitivity to Oxidative Stress. Mol. Cell. Biol..

[46] Nakajima H., Itakura M., Kubo T., Kaneshige A., Harada N., Izawa T., Azuma Y.-T., Kuwamura M., Yamaji R., Takeuchi T. (2017). Glyceraldehyde-3-Phosphate Dehydrogenase (GAPDH) Aggregation Causes Mitochondrial Dysfunction during Oxidative Stress-Induced Cell Death. J. Biol. Chem..

[47] Wang S., Liang Y., Dai C. (2022). Metabolic Regulation of Fibroblast Activation and Proliferation during Organ Fibrosis. Kidney Dis.

[48] Delgado M.E., Cárdenas B.I., Farran N., Fernandez M. (2021). Metabolic Reprogramming of Liver Fibrosis. Cells.

[49] Bos S., Laukens D. (2020). Metabolic Modulation during Intestinal Fibrosis. J. Dig. Dis..

[50] Satyanarayana G., Turaga R.C., Sharma M., Wang S., Mishra F., Peng G., Deng X., Yang J., Liu Z.-R. (2021). Pyruvate Kinase M2 Regulates Fibrosis Development and Progression by Controlling Glycine Auxotrophy in Myofibroblasts. Theranostics.

[51] Han H., Zhang Y., Peng G., Li L., Yang J., Yuan Y., Xu Y., Liu Z.-R. (2021). Extracellular PKM2 Facilitates Organ-Tissue Fibrosis Progression. iScience.

[52] Jin E.S., Lee M.H., Malloy C.R. (2020). Divergent Effects of Glutathione Depletion on Isocitrate Dehydrogenase 1 and the Pentose Phosphate Pathway in Hamster Liver. Physiol. Rep..

[53] Jin E.S., Lee M.H., Murphy R.E., Malloy C.R. (2018). Pentose Phosphate Pathway Activity Parallels Lipogenesis but Not Antioxidant Processes in Rat Liver. Am. J. Physiol. Endocrinol. Metab..

[54] Almazroo O.A., Miah M.K., Venkataramanan R. (2017). Drug Metabolism in the Liver. Clin. Liver Dis..

[55] Horio F., Shibata T., Naito Y., Nishikimi M., Yagi K., Yoshida A. (1993). L-Gulono-Gamma-Lactone Oxidase Is Not Induced in Rats by Xenobiotics Stimulating L-Ascorbic Acid Biosynthesis. J. Nutr. Sci. Vitaminol..

[56] Darr D., Combs S., Pinnell S. (1993). Ascorbic Acid and Collagen Synthesis: Rethinking a Role for Lipid Peroxidation. Arch. Biochem. Biophys..

[57] Karna E., Szoka L., Huynh T.Y.L., Palka J.A. (2020). Proline-Dependent Regulation of Collagen Metabolism. Cell. Mol. Life Sci..

[58] Chang M.-L., Yang S.-S. (2019). Metabolic Signature of Hepatic Fibrosis: From Individual Pathways to Systems Biology. Cells.

[59] Smith-Cortinez N., van Eunen K., Heegsma J., Serna-Salas S.A., Sydor S., Bechmann L.P., Moshage H., Bakker B.M., Faber K.N. (2020). Simultaneous Induction of Glycolysis and Oxidative Phosphorylation during Activation of Hepatic Stellate Cells Reveals Novel Mitochondrial Targets to Treat Liver Fibrosis. Cells.

[60] Sánchez-Valle V., Chávez-Tapia N.C., Uribe M., Méndez-Sánchez N. (2012). Role of Oxidative Stress and Molecular Changes in Liver Fibrosis: A Review. Curr. Med. Chem..

[61] Luangmonkong T., Suriguga S., Mutsaers H.A.M., Groothuis G.M.M., Olinga P., Boersema M. (2018). Targeting Oxidative Stress for the Treatment of Liver Fibrosis. Rev. Physiol. Biochem. Pharmacol..

[62] Unsal V., Cicek M., Sabancilar İ. (2021). Toxicity of Carbon Tetrachloride, Free Radicals and Role of Antioxidants. Rev. Environ. Health.

[63] Staňková P., Kučera O., Lotková H., Roušar T., Endlicher R., Cervinková Z. (2010). The Toxic Effect of Thioacetamide on Rat Liver in Vitro. Toxicol. Vitr..

[64] Read A.D., Bentley R.E., Archer S.L., Dunham-Snary K.J. (2021). Mitochondrial Iron-Sulfur Clusters: Structure, Function, and an Emerging Role in Vascular Biology. Redox Biol..

[65] Ezhilarasan D. (2021). Mitochondria: A Critical Hub for Hepatic Stellate Cells Activation during Chronic Liver Diseases. Hepatobiliary Pancreat. Dis. Int..

[66] Ban D., Hua S., Zhang W., Shen C., Miao X., Liu W. (2019). Costunolide Reduces Glycolysis-Associated Activation of Hepatic Stellate Cells via Inhibition of Hexokinase-2. Cell. Mol. Biol. Lett..

[67] Yu H., Zhu J., Chang L., Liang C., Li X., Wang W. (2021). 3-Bromopyruvate Decreased Kidney Fibrosis and Fibroblast Activation by Suppressing Aerobic Glycolysis in Unilateral Ureteral Obstruction Mice Model. Life Sci..

[68] Zhang A., Qian F., Li Y., Li B., Yang F., Hu C., Sun W., Huang Y. (2023). Research Progress of Metformin in the Treatment of Liver Fibrosis. Int. Immunopharmacol..

[69] Musso G., Cassader M., Paschetta E., Gambino R. (2017). Thiazolidinediones and Advanced Liver Fibrosis in Nonalcoholic Steatohepatitis: A Meta-Analysis. JAMA Intern. Med..

