# Supplementary material for: Fibrosis Development Linked to Alterations in Glucose and Energy Metabolism and Prooxidant–Antioxidant Balance in Experimental Models of Liver Injury
Source: Antioxidants (Basel). 2023 Aug 12;12(8):1604. doi: 10.3390/antiox12081604 (PMC10451385; doi:10.3390/antiox12081604)
Supplement: Supplementary file 1 [file antioxidants-12-01604-s001.zip › antioxidants-2532103-supplementary.pdf]

Fibrosis Development Linked to Alterations in Glucose and Energy Metabolism and Prooxidant–Antioxidant Balance in Experimental Models of Liver Injury

Dmitry S. Semenovich [1,*], Nadezda V. Andrianova [1], Ljubava D. Zorova [1,2], Irina B. Pevzner [1,2], Polina A. Abramicheva [1], Andrey V. Elchaninov [3], Olga V. Markova [1], Aleksandra S. Petrukhina [4], Dmitry B. Zorov [1,2] and Egor Y. Plotnikov [1,2,*]

Supplementary Tables

Supplementary Table S1. The number of rats in each experimental group.

Assay	Experimental groups				
	Control (intact rats)	TAA	CCl_4	BDL	I/R
Morphological studies	8	6	6	6	6
Biochemical analysis of the blood	8	6	6	6	7*
Hydroxyproline measurement	6	6	6	6	6
Determination of substrates and products of glucose metabolism	6	6	5	5	7*
The activity of the enzymes of glycolysis and the pentose phosphate pathway	6	6	6	6	6
The activity of the enzymes of the glucuronic pathway	6	5	5	5	6
PDHC and Krebs cycle enzymes activity	6	6	6	4	5
Respiratory activity of mitochondria	6	5	6	4	4
Western blot	4	4	4	4	4
RT-PCR	3	5	4	3	3*
Oxidative stress parameters	5	4	4	4	5

*the value of "n" may be less than the number of rats indicated in the table for the I/R group due to the presence of outliers estimated by the Grubbs test.

Supplementary Figures

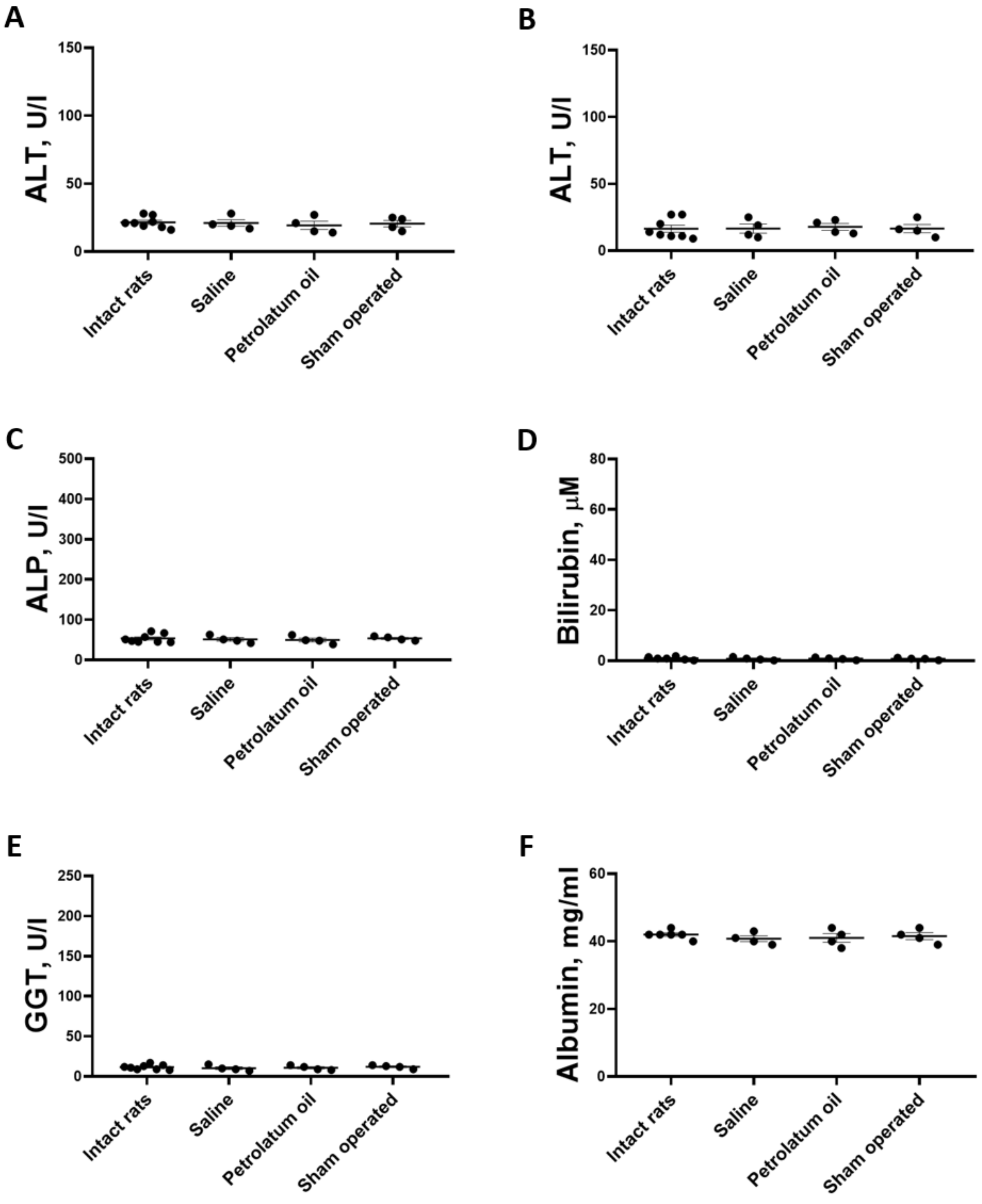

Supplementary Figure S1. Liver injury markers in control groups. (A) Activity of AST in blood serum. (B) Activity of ALT in blood serum. (C) Activity of ALP in blood serum. (D) Bilirubin concentration in blood serum. (E) GGT activity in blood serum. (F) Albumin concentration in blood serum. The number of rats n≥4 in all experimental groups.

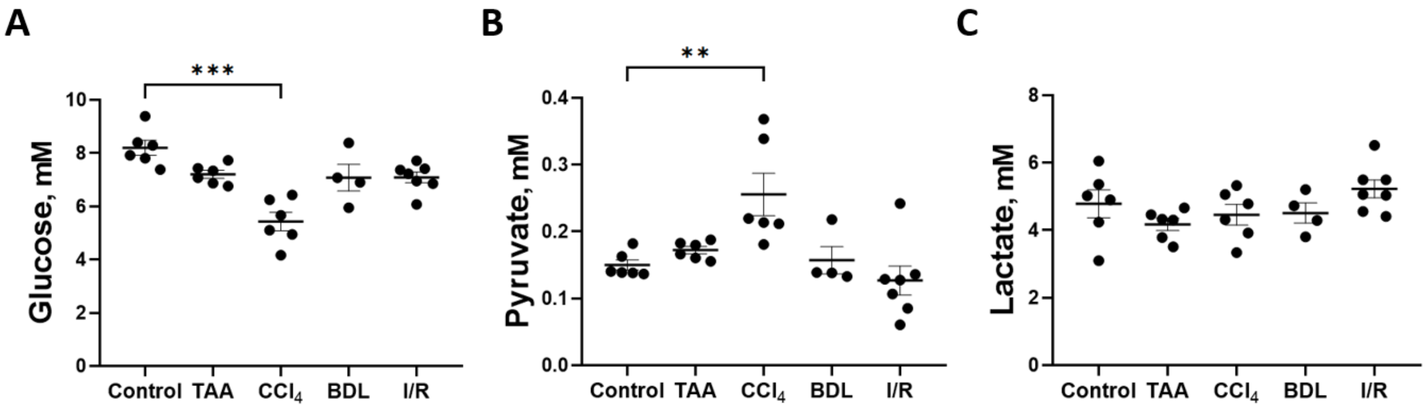

Supplementary Figure S2. Glucose and its metabolites in the whole blood of rats with various liver lesions. (A) Glucose concentration. (B) Pyruvate concentration. (C) Lactate concentration. ** $p < 0.01$, *** $p < 0.001$ (one-way ANOVA).

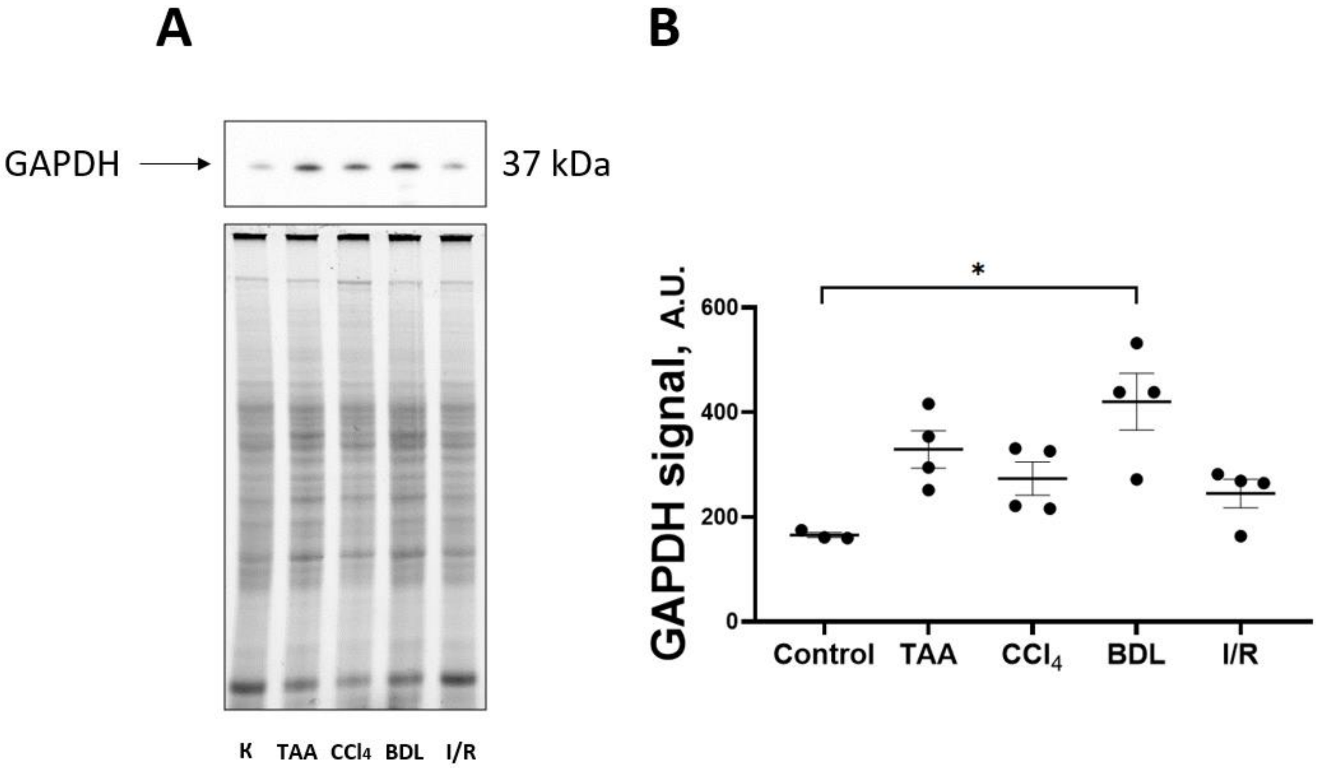

Supplementary Figure S3. GAPDH content in rat liver tissue with various injuries. (A) Representative Western blot bands. Total protein loading was estimated using a stain-free imaging technique. (B) Quantification of Western blot. *$p<0.05$ (one-way ANOVA).

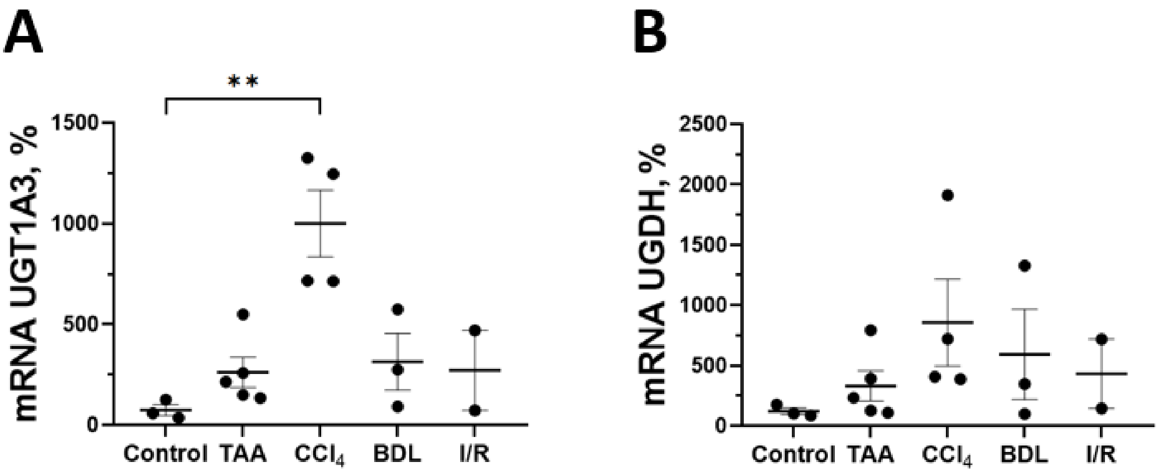

Supplementary Figure S4. Expression of enzymes of the glucuronidation pathway. (A) mRNA expression of UDP-glucuronosyltransferase 1-3 (isoform UGT1A3). (B) UDP-glucose dehydrogenase expression. **$p<0.01$ (one-way ANOVA).
